# In-Situ Exploration of Phytic Acid-Mediated Supramolecular Self-Assembly and Gelation

**DOI:** 10.1021/acs.analchem.5c05630

**Published:** 2026-01-30

**Authors:** Yu-Sheng Yen, Chia-Wei Zhang, Wei-Tsung Chuang, Chun-Fu Chang, Hirotsugu Hiramatsu, Hsin-Yun Hsu

**Affiliations:** † Department of Applied Chemistry and Institute of Molecular Science, 34914 National Yang-Ming Chiao-Tung University, Hsinchu 300093, Taiwan; ‡ National Synchrotron Radiation Research Center (NSRRC), Hsinchu 300092, Taiwan; § Center for Emergent Functional Matter Science, National Yang-Ming Chiao-Tung University, Hsinchu 300093, Taiwan

## Abstract

Molecular gels emerge as a promising type of material owing to their high tunability for versatile applications. However, there are still gaps in our mechanistic understanding of the molecular assembly and its consequent physicochemical properties. Additionally, most of the methods for hydrogel characterization require pretreatment, which makes it difficult to verify the exact fibril structures of the hydrogels and the intermolecular interactions involved. Herein, we employed Raman spectroscopy, a technique frequently used to verify intermolecular forces, to revisit the dynamic molecular self-assembly of guanosine monophosphate in the acidic milieu. In addition to the confirmed presence of G-quadruplexes in the hydrogel, a previously unidentified peak was discovered in the low wavenumber region (∼96–110 cm^–1^), potentially referring to the lattice packing of G-tetrads. The phytic acid (PA)-mediated formation of G-quadruplex-based hydrogels consisted of a bifibre-bundle structure, resulting in higher mechanical strength. Furthermore, we found that monitoring the P–OH stretching mode (850–860 cm^–1^) and the water-associated hydrogen bonding in the high wavenumber region (>2000 cm^–1^) revealed that, at low PA concentrations, PA molecules mainly act as cross-linkers between G-quadruplex fibrils. When the concentration increases, the aggregation of excess PA may form clusters to support the gel void, providing extra mechanical strength. This study establishes a methodology to noninvasively resolve the supramolecular hydrogel self-assembly.

## Graphical abstract

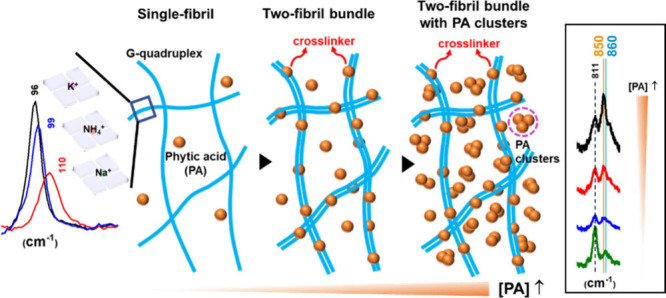

## Introduction

Molecular self-assembly has emerged as a fundamental paradigm in materials science and biotechnology, with extensive research focusing on molecular design principles, functional integration, size control, and mechanical property optimization. Large biomolecular systems, particularly peptides and proteins, have demonstrated remarkable self-assembly capabilities that enable diverse applications, including stimuli-responsive materials for regenerative medicine, biosensors, and antimicrobial coatings.^1,2^ Similarly, DNA origami^3^ and protein cages^4^ have shown extraordinary precision in creating complex three-dimensional architectures for nanoscale applications. However, as the molecular complexity increases, significant challenges arise in synthesis, purification, characterization, and scalable production. The intricate preparation procedures, often requiring specialized equipment and conditions coupled with the inherent instability of many large biomolecules under processing conditions, limit their practical implementation.

This complexity paradox has driven researchers toward a reductionist approach: identifying the essential structural motifs within large self-assembling molecules and designing minimal molecular architectures that retain equivalent functionality while offering superior processability and tunability. Low-molecular-weight gelators (LMWGs) exemplify this strategy, representing a class of small molecules (typically <3000 Da) capable of forming three-dimensional networks that immobilize solvents at remarkably low concentrations (often ≤0.5 wt %).^5,6^ These supramolecular gels offer distinct advantages over their macromolecular counterparts, including synthetic accessibility, structural simplicity, and enhanced responsiveness to external stimuli. Among LMWGs, nucleobase derivatives have attracted particular attention due to their unique combination of structural features: heteroatom-rich frameworks that facilitate hydrogen bonding, planar aromatic systems enabling π–π stacking interactions, and coordination sites for metal–ligand binding. This multifaceted binding capacity allows for sophisticated hierarchical assembly processes that can be systematically modulated through a molecular design. Despite growing interest in multicomponent nucleobase-derived gel systems,^7,8^ significant knowledge gaps persist in predicting and controlling gelator organization within fiber networks and understanding the relationship between molecular-level structural changes and macroscopic rheological properties.^9^ Bridging this structure–function gap remains crucial for advancing rational design principles and unlocking the full potential of LMWG-based materials in applications ranging from biomedical devices to responsive soft robotics.

The characterization of the hydrogel is of great importance to explore its physicochemical properties, the gelation mechanism, and the interactions involved. In particular, the interpretation of the structures can provide clues for the molecular design and subsequent applications. Rheological measurements are used to understand the physical properties of gels such as mechanical strength and viscoelasticity,^10–12^ whereas scanning electron microscopy (SEM)^13,14^ and powder X-ray diffraction (PXRD)^14,15^ are often employed to respectively observe the gel morphologies and the crystalline phases. Nonetheless, in many studies, gel samples were freeze-dried,^16^ or diluted^17^ for subsequent investigation. The requirement for sample pretreatment before observation has been a major concern. The freeze-drying process sublimates the solvents, which could alter the texture of gels,^18^ and dilution results in changes in the concentration of the components. Neither of these guarantees that the original gel properties could be observed. Alternatively, scientists have employed FT-IR to identify the characteristic peak shifts to confirm the chemical bonding of the hydrogels.^15,16,19^ Although FT-IR provides information on the intermolecular interactions of gels, hydrogel, as a material rich in water content, contributes to large water signals that could interfere with resolving the IR spectra.^20–22^ Similar issues also occur in ^1^H NMR spectra analysis^15,23,24^ by the presence of strong water signals, which could cause spectral distortion and mask the nearby weak solute signals.^20–22^ Additionally, extreme dynamic heterogeneity in molecular mobility creates broadened signals, and cross-link characterization remains elusive due to the low signal abundance and exchange dynamics in such LMWG systems since they often lack distinct chemical signatures. Alternatively, simply increasing the gelator concentration to improve NMR sensitivity would fundamentally alter the gel properties, making it difficult to employ in situ to correlate the spectroscopic data with the actual gel behavior under the conditions of interest.

Therefore, suitable approaches are critical for the exploration of hydrogel structures and intermolecular interactions. Raman spectroscopy has been a useful technique that is complementary to the IR spectra for chemical bonding identification.^25^ This nondestructive approach employs the laser to pass through the analyte and is dependent on the scattering of photons resulting from the molecular-level energy transfer. The high specificity of the scattering provides the information on atomic and molecular structures, with the advantage that it can potentially assist in elucidating the structural and conformational changes in a range of complex fluid systems and is compatible with aqueous solutions.^15,26^ Raman-based techniques have been used to explore molecular interactions in different phases.^27,28^ Recent analysis with Raman spectroscopy in gel-based systems has focused primarily on observing changes in free and confined water by investigating the high-frequency vibrational bands in the spectra.^29,30^

In this study, we employed Raman spectroscopy along with UV–vis, SAXS, and rheological analysis to explore acid-mediated guanosine monophosphate (GMP) gelation. It has been proposed that, under acidic pH, protonation of the negatively charged phosphate group of GMP can reduce the electrostatic repulsion between the molecules, facilitating the assembly of G-tetrads and the G-quadruplexes fibril formation.^16,31^ Instead of conventional strategies which introduced borate derivatives to form the covalent borate diester backbond for the enhancement of gel stability,^32,33^ we employed phytic acid (PA) to initiate gelation in the presence of K^+^. PA is the hexaphosphate of inositol with multiple H-bonding sites, which potentially may serve as a cross-linker. Nonetheless, it remains highly charged over a wide range of pH; it is also interesting how such a large molecule (with a diameter of ∼10 Å) assembles and stabilizes the G-quadruplexes and the assembly into the gel network. We aim to understand the molecular arrangement of protonated and deprotonated nucleotides, ions, void space, and packing and aggregates in the network-level of assembly that may contribute to the varied bulk properties of these hydrogels.

## Results and Discussion

### The K^+^-Coordinated, PA-Mediated GMP-Based Hydrogel Synthesis

The PA-mediated GMP-based hydrogel consisted of three components: K^+^, PA, and GMP. KCl as the potassium source was first mixed with GMP to enable G-quadruplex formation, followed by the addition of PA for gelation. The tube inversion test was utilized to efficiently screen whether gelation occurred (Figure S1). Interestingly, the [KCl-GMP-PA] hydrogel revealed high turbidity (Figure S2), using KOH instead of KCl as the potassium source for hydrogel preparation (i.e., [KOH-GMP-PA]), and the gel appeared to be transparent (Figure 1a). The [PA] and [KOH] concentration effects on the [KOH-GMP-PA] gel system were then investigated by UV-vis measurements (Figure S3). The overall absorbance of the tested samples was lower than that of the pure GMP solution, which could refer to the hypochromic effect contributed by the π-π stacked G-tetrads. The strong interaction between stacked nitrogenous bases reduces the UV–vis absorption. The lowest absorbance was identified respectively at [PA] = 60 mM and [KOH] = 364.8 mM, implying that the stable GMP-assembled G-quadruplex could be formed at this optimized condition. By varying [GMP] from 0 to 120 mM along with fixed values of [KOH] = 364.8 mM and [PA] = 60 mM, the UV-vis spectra normalized at 252 nm are shown in Figure 1b. A decrease in the absorption of isolated monomeric GMP at 265–285 nm and an increase in the absorption of the delocalization feature at 290–310 nm, relative to the pure GMP solution, could be observed in all tested samples, indicating the likely G-tetrad structure formation during gelation.^34^

**Figure 1:**
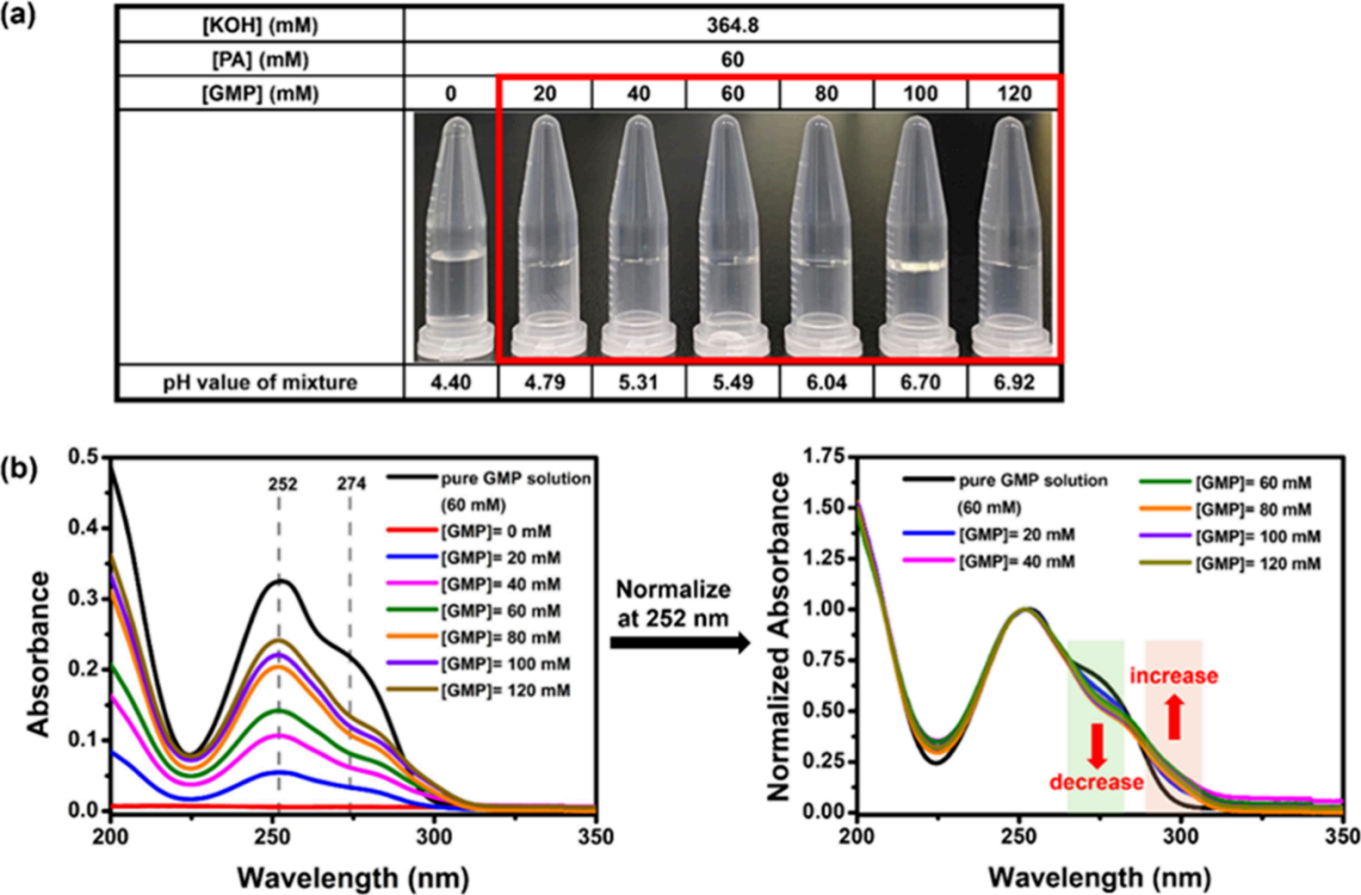
[GMP] concentration effect on [KOH-GMP-PA] hydrogel formation: (a) photographic images of tube inversion test (red square indicated the gel formation); (b) UV–vis spectra (left) and the spectra normalized at 252 nm (right).

In addition to the difference in turbidity between [KOH-GMP-PA] and [KCl-GMP-PA] hydrogels, the mechanical strengths of the two were also distinct, according to the rheological measurements. In such analysis, storage modulus (*G*′) indicates the amount of energy stored in an elastic material, suggesting the elasticity of the sample, whereas the loss modulus (*G*″) represents the energy dissipated in heat during the deformation of materials and can be used to quantify the viscous behavior. As shown in Figure 2 and Figure S4, the characteristic of the hydrogels (elastic solid) was confirmed. *G*′ was larger than *G*″, and the calculated loss factor (tan δ = *G*″/*G*′) in all tested samples was <1. Nonetheless, stronger mechanical strength was found in [KCl-GMP-PA] hydrogels (with maximum *G*′ > 10^4^ Pa) (Figure 2a) than in [KOH-GMP-PA] hydrogel (with maximum *G*′ ≈ 10^3^ Pa) (Figure 2b), regardless of the concentration of GMP, PA, or K^+^. The frequency-dependent power-law behavior of *G*′ and *G*″ yields a fractional exponent β, typically ranging from 0 to 1.^35^ A lower β value (closer to 0) reflects a more elastic-dominated response, whereas a higher β value (closer to 1) corresponds to viscous-dominated characteristics. As shown in Tables S1 and S2, our measured β values lie between 0.02 and 0.33, indicating a predominantly solid-like gelation behavior with weak power-law dependence for the hydrogels.

**Figure 2:**
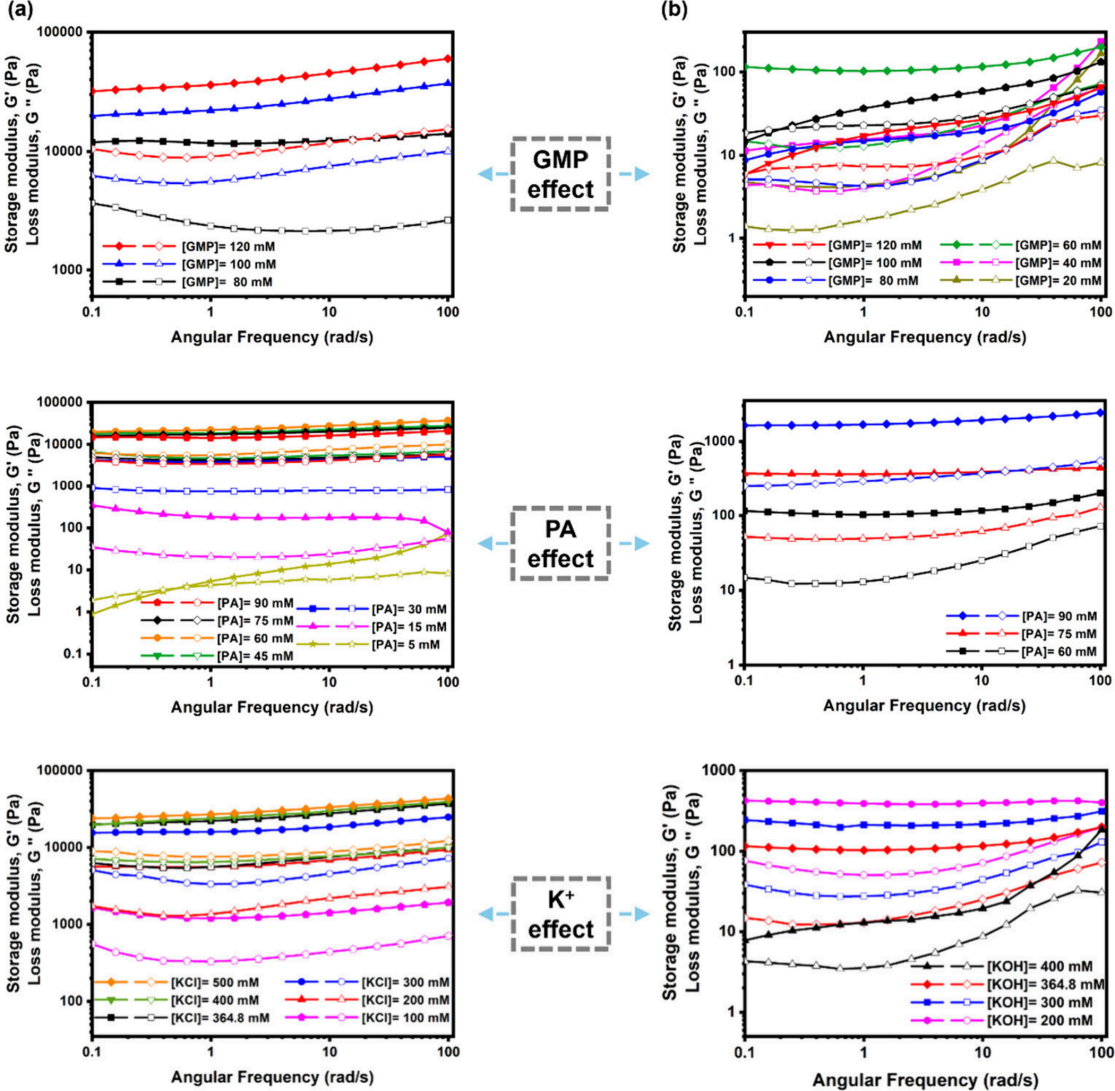
Rheological measurements of (a) [KCl-GMP-PA] and (b) [KOH-GMP-PA] hydrogels ([GMP], [PA], and [K^+^] concentration effects were respectively compared (top, middle, and bottom); solid symbol represents *G*′; open symbol represents *G*″)).

### The pH Effect on the Fibril Structure of the Gel

The differences in the turbidity and mechanical strength of the [KCl-GMP-PA] and [KOH-GMP-PA] hydrogels could be attributed to the distinct pH in the two systems. KCl is a neutral salt, and KOH acts as a strong inorganic base. In the presence of the same amount of three components (e.g., [K^+^] = 364.8 mM, [GMP] = 100 mM, and [PA] = 60 mM), the pH of [KCl-GMP-PA] (Figure S1­(a)) and [KOH-GMP-PA] (Figure 1a) hydrogels were 1.20 and 6.70 respectively, which were distinct from each other. As known previously, pH could affect the protonation/deprotonation of guanine on GMP, which, in turn, leads to the structural changes of G-tetrads. The hydrogen bonding between N7 and N2′H forms at pH 3–5, which is favorable for the generation of the G-quadruplex structure. Alternatively, at pH <1.9, the N7 position is protonated (the p*K*_a_ of the N7 position on the guanine is 1.9). Hydrogen bonding between N7 and N2′H is relatively unfavorable to form, and whether the G-tetrad structure remains stable is unclear. Nonetheless, the negatively charged phosphate group can also be protonated to reduce the electrostatic repulsive force of the overall system.

The rheological analysis and SAXS/WAXS techniques were employed to understand the effect of pH on the macroscopic and nanoscale structural changes of the gel. Hydrogel [KCl-GMP-PA] was prepared at pH 1, 2, and 3 respectively (Figure 3a). The turbidity and mechanical strength (Figure 3b) of the gel samples increased with decreasing pH (increasing [PA]). As shown in SAXS/WAXS profiles (Figure 3c), there was a broad characteristic peak in the middle *q* region in all tested samples, corresponding to the longest distance in the transverse plane (*x*–*y* plane) of the bundle formed by multiple G-quadruplex fibrils. As pH decreased, this characteristic peak gradually moved toward the low *q* direction (*q* = 0.22, 0.17, and 0.15 Å^–1^ for pH 3, 2, and 1), which indicated an increase in *d*-spacing size (q= 28.6, 37.0, and 41.9 Å for pH 3, 2, and 1). We have known that the outer diameter of the single fibril in the [KCl-GMP-HCl] hydrogel was about 14.3 Å via molecular modeling (Figure S5). As revealed by SAXS/WAXS profile of PA-mediated GMP-based hydrogel [KCl-GMP-PA] at pH 3, a two-fibril bundle structure was thus proposed (*d* = 28.6, which is twice the single fibril diameter). Further lowering the pH to 1, the *d*-spacing of the experimental characteristic peak was 41.9 Å (Figure 3d), which is approximately three times the outer diameter (14.3 Å). There was a tendency for the G-quadruplex fibrils to form a bundle in a hexagonal arrangement. It was likely that the increase in gel mechanical strength, as well as the turbidity, was a result of multiple fibrils that were aggregated to form the bundles in the gel matrix as the pH decreased.

**Figure 3:**
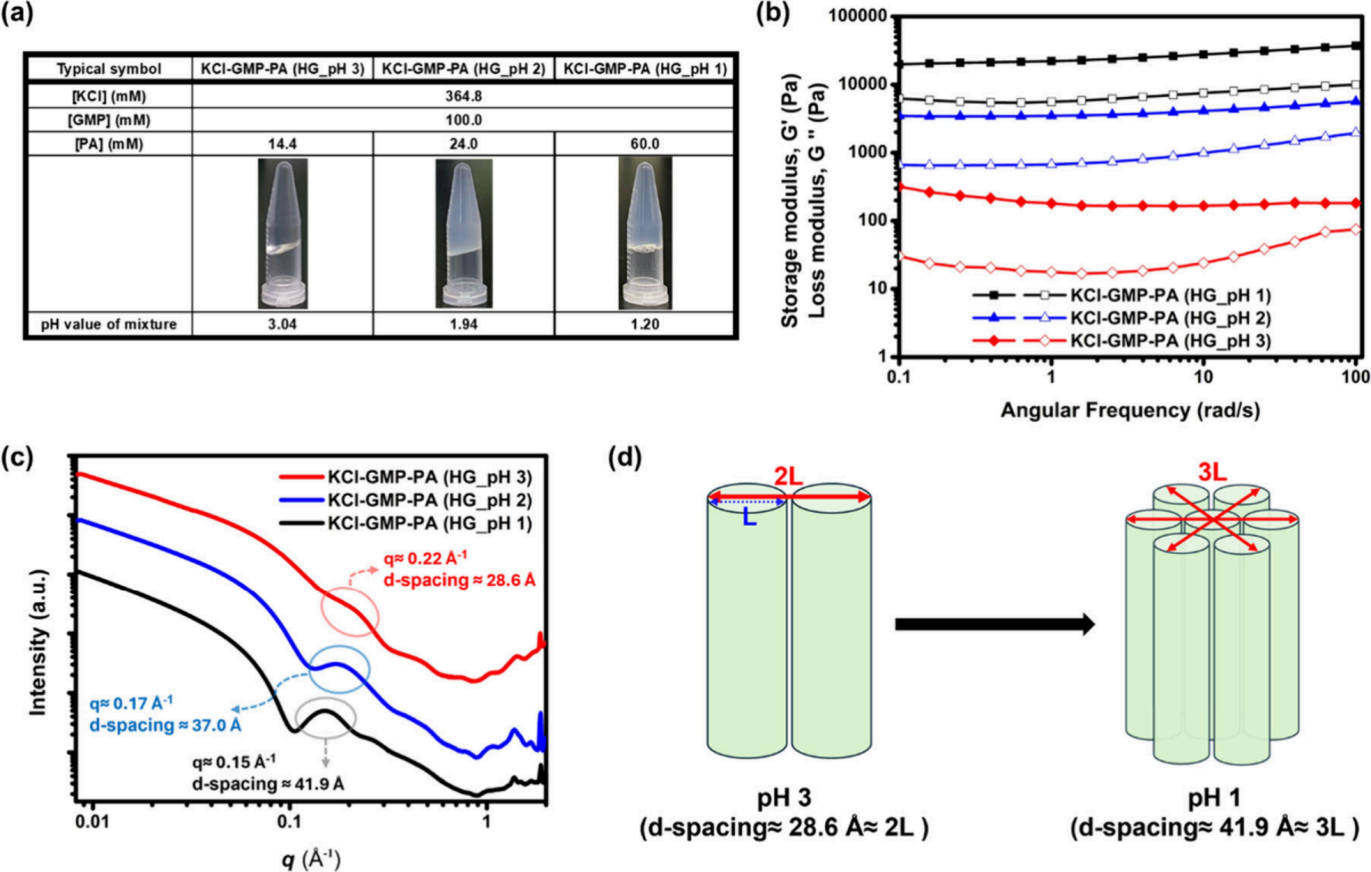
(a) Photographic images of the tube inversion test (gel formed in all three conditions), (b) rheological measurement, and (c) SAXS/WAXS profiles of [KCl-GMP-PA] hydrogels at pH 1, 2, and 3. (d) Proposed schematic diagram of hexagonal bundle formation comprised of G-quadruplex fibrils (*L* ≈ 14.3) in [KCl-GMP-PA] hydrogel at pH 1.

### Molecular Interactions of Acid-Triggered GMP Self-Assembly

As pH has been known to affect the G-tetrad formation and the fibril structure of the PA-mediated GMP-based hydrogels, to simplify the system for investigation, all samples were kept at pH 3. In addition to PA, HCl and phosphoric acid (H_3_PO_4_) were included as controls to enable better clarification of the interactions between PA and the G-quadruplex fibrils. Also, both KCl and KOH as the potassium sources were employed for comparison (abbreviated as [KCl-GMP-Acids] and [KOH-GMP-Acids] hydrogels; “Acids” referred to PA, H_3_PO_4_, or HCl). From the rheological tests, we noticed that K^+^ was critical as the coordinating metal. The mechanical strength is relatively weak (*G*′ ≈ 35 Pa at 0.1 rad/s) in the absence of K^+^ (Figure S6­(a)). With KCl, the *G*′ value reached about 200–350 Pa at 0.1 rad/s (Figure S6­(b)) under all three acid conditions. It was noteworthy, however, with KOH as the potassium source, although the mechanical strength of gel could still have a *G*′ value (at 0.1 rad/s) of up to 300 Pa using PA, in the cases of H_3_PO_4_ and HCl, *G*′ decreased to about 100 and 50 Pa, respectively (Figure S6­(c)). Obviously, there were different molecular interactions in [KCl-GMP-Acids] and [KOH-GMP-Acids] hydrogels. How PA may interact with the G-quadruplex fibrils to maintain the gel texture and the resulting mechanical strength shall be discussed later.

UV-vis spectra were measured to reconfirm the G-tetrad formation among these conditions. For the samples without K^+^ (Figure S7­(a)), although a slight decrease in *A*_252 nm_ (from 0.48 to 0.34) was observed compared to the pure GMP spectrum, normalized spectra showed no much differences, indicating GMP molecules may only have limited amorphous aggregation of monomers that reduce absorbance. In contrast, in the spectra of [KCl-GMP-Acids] and [KOH-GMP-Acids] hydrogels, the normalized spectra showed characteristic absorption of G-tetrad formation (Figure S7b,c), signifying the essential role of K^+^ for stabilizing the G-tetrad structure. An obvious decrease from 0.48 to 0.25 at *A*_252 nm_ further suggested that the hypochromic effect occurred as a result of π–π stacked G-tetrad to form a G-quadruplex.

Raman difference spectra (Figure 4) were then performed to detect any differences other than the intensity. The coefficients (see the supporting materials and methods, including eq S1, in the Supporting Information) were adjusted to minimize the difference spectrum. Since the gel samples were controlled at pH 3, the reference for this was the GMP solution at pH 3 (KOH was used for pH adjustment, and 40 mM GMP was chosen to make the final solution). Subtracting the reference from the gel samples gives the difference in the molecular interactions between the gel and solution under the same pH environment. There are positive peaks at 666, 811, and 1337 cm^–1^ in the difference spectra for [GMP-Acids], [KCl-GMP-Acids], and [KOH-GMP-Acids] hydrogels, which represent new emergent features. The Raman shifts of 666 and 1337 cm^–1^ are assigned as changes in sugar group conformation on the GMP molecules.^36,37^ Regarding the prominent peak at 811 cm^–1^, previous studies proposed that it could be the ″phosphodiester group frequency″ (O–P = O symmetric stretching mode).^38^ This small characteristic peak was also observed in both pure GMP and the GMP aqueous solutions at pH 3 (blue peak in Figure S8). A remarkable Raman hyperchromic effect occurred at this peak upon gelation. This could be due to the hydrogen bond formation between the phosphate oxygen and the amino group of a guanine base three bases away on the helix (i.e., the symmetric stretching vibration of the C–O–P=O**···**H group).^36,39^

**Figure 4:**
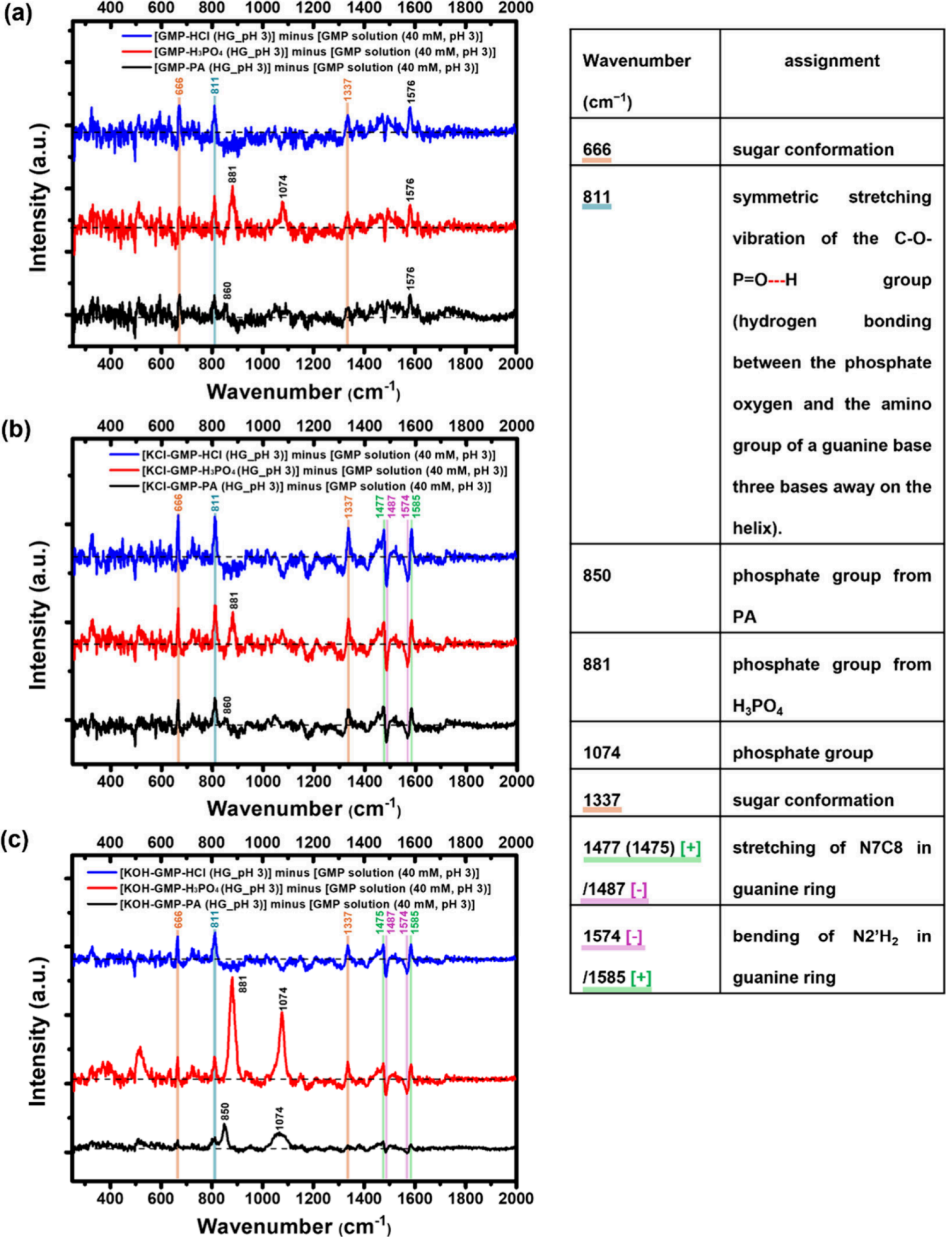
Raman difference spectra to explore molecular interactions involved in (a) [GMP-Acids], (b) [KCl-GMP-Acids], and (c) [KOH-GMP-Acids] hydrogels (“Acids” referred to PA, H_3_PO_4_, or HCl; Raman spectra of PA, H_3_PO_4_ and HCl solution referred to Figure S12; [Hydrogel (HG) sample] minus [GMP solution (40 mM, pH 3)] is intended to identify newly appeared or disappeared peaks (any differences other than intensity); thus, the coefficient is chosen to minimize the difference spectra).

Additional characteristic peaks in the fingerprint region (1470–1590 cm^–1^) of the [KCl-GMP-Acids] and [KOH-GMP-Acids] hydrogel difference spectra were identified. There was a combination of positive 1477(1475) cm^–1^ and negative 1487 cm^–1^ features observed, where ∼1487 cm^–1^ is assigned as the N7–C8 stretching mode of guanine five-membered ring.^40,41^ Once the Hoogsteen hydrogen bonding in G-tetrad structure is formed, it results in the peak downshift ∼6–9 cm^–1^, and thus, a positive feature at ∼1477 cm^–1^ could be found in the difference spectra.^42^ The other was a set of negative 1574 cm^–1^ and positive 1585 cm^–1^. Raman shift at 1574 cm^–1^ is identified to be the N2′H_2_ bending mode of the C2 connection on the guanine six-membered ring.^41^ Once the hydrogen bond between the N7 and N2′H of the G-tetrad structure is established, it would lead to the peak upshift to ∼1585 cm^–1^ position.^42^ Positive, negative, negative, and positive characteristic peaks obtained in this region of the Raman difference spectrum, along with UV-vis measurements (Figure S7) provided evidence for the formation of the G-quadruplex fibril structure in the K^+^-containing gel systems, whereas in the control sample [GMP-Acids] (without K^+^) these features were hardly observed.

Besides the fingerprint region, Raman spectra of different acid-mediated hydrogels were also measured in the relatively unexplored low wavenumber region (<250 cm^–1^) (Figure 5). In contrast to the control solution that showed no peaks (Figure 5a), [GMP-Acids] gels had a weak signal around 110 cm^–1^ (Figure 5b), and both [KCl-GMP-Acids] and [KOH-GMP-Acids] hydrogels exhibited sharp peaks around 96 cm^–1^ (Figure 5c and 5d). To exclude the heating effect, Raman spectra were acquired under continuous irradiation or varying laser powers and showed no band shifts (Figure S9­(a) and S9­(b)). We suspected it may be a feature of the association of the four GMP molecules in the G-tetrad plane through hydrogen bonding, or it could be a signal of π–π stacking between the G-tetrad planes upon K^+^ addition, to clarify the exact causes, the central K^+^ was replaced by either Na^+^ or NH_4_^+^ in hydrogels to examine the peak shifts (Figure 5e). By variation of the central cations, the obvious peak shifts were observed. Raman shifts could be affected by packing of the lattice. The larger lattice distance has a lower frequency. Na^+^ is coordinated within the plane of a G-tetrad, while K^+^ and NH_4_^+^, with larger ionic radii, are positioned between two stacked layers, resulting in a larger interlayer distance. The cation-specific characteristic peaks of 110 cm^–1^ (for Na^+^), 99 cm^–1^ (for NH_4_^+^), and 96 cm^–1^ (for K^+^) in the Raman spectra were found, corresponding to the G-tetrad interlayer distance differences. Increasing the concentration of GMP or K^+^ in [KCl-GMP-HCl] revealed an enhanced peak intensity at 96 cm^–1^ (Figure S10­(a) and S10­(b)), which further confirmed this K^+^-coordinated G-tetrad lattice and indicated more G-tetrad assembly as expected. The pH changes, which potentially influence the hydrogen bonding during G-tetrad assembly, resulted in changes of peak intensity rather than peak shifts in the [KCl-GMP-HCl] hydrogel (Figure S10­(c)). Different isotopic deuterium-substituted GMP molecules were also employed to monitor the Raman peak changes (Figure 5f). We did not observe significant peak shifts in the spectra, indicating that this newly identified characteristic Raman peak is mainly influenced by the different π–π stacking modes of G-tetrad planes and can serve as a distinct marker of G-tetrad assembly. This assignment is further supported by polarized Raman measurements (Figure S9­(c)). Since π–π stacking is highly directional, we found that the intensity of the ∼ 96–110 cm^–1^ band is sensitive to a particular polarization (vertical polarization in our experimental configuration). To confirm that the ∼96–110 cm^–1^ band is specific to the π–π stacking modes of the G-tetrad, we performed control experiments by replacing GMP with AMP, CMP, or UMP. The ∼96–110 cm^–1^ band completely vanishes in the Raman spectra of AMP, CMP, and UMP (Figure S10­(d)), confirming its specificity to G-tetrad structures. We also performed temperature-controlled Raman measurements and found that the structure of GMP assembly disassembly can be modulated reversibly by changing the temperature (Figure S11).

**Figure 5:**
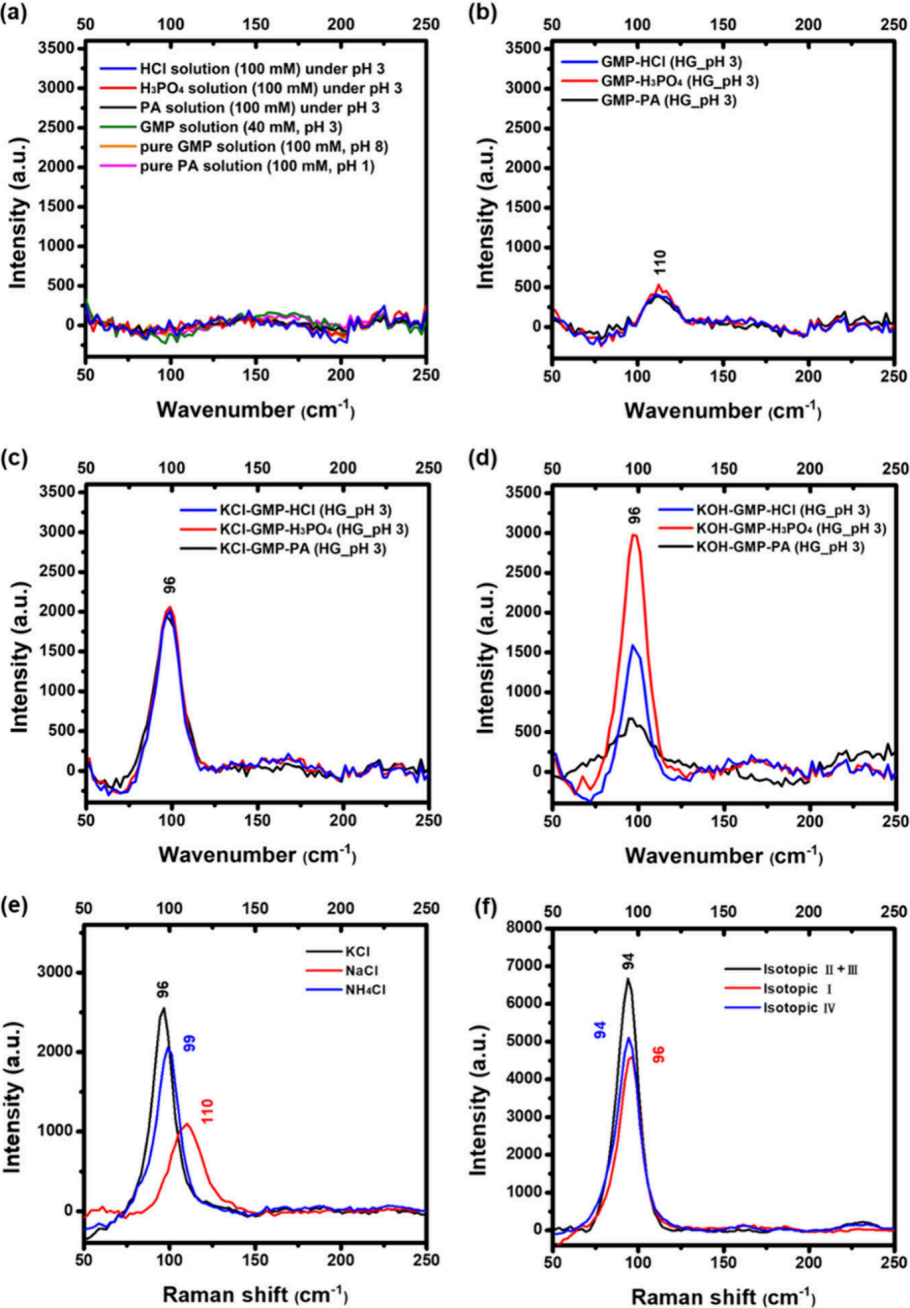
Raman spectra in the low wavenumber region (<250 cm^–1^): (a) control solution: pure PA solution (pH 1), pure GMP solution (pH 8), GMP solution adjusted to pH 3 by HCl, as well as PA, H_3_PO_4_ and HCl solution adjusted to pH 3 by KOH, (b) [GMP-Acids], (c) [KCl-GMP-Acids], (d) [KOH-GMP-Acids] hydrogels (“Acids” referred to PA, H_3_PO_4_, or HCl), (e) [MCl-GMP-HCl] (pH 3) hydrogels (“MCl” referred to KCl, NaCl, or NH_4_Cl) and (f) different isotopic deuterium-substituted GMP molecules in [KCl-GMP-HCl] (pH 3) hydrogel.

Since water is the major content in the hydrogels, investigating water molecules in these hydrogels provides additional insights. Hamaguchi et al. employed the Raman spectrometry to study water molecules in the temperature range from −23 °C to 45 °C. By multivariate curve resolution with alternate least-squares (MCR-ALS) and hypothetical addition multivariate analysis with numerical differentiation (HAMAND) methods,^43^ the results showed that structured hydrogen-bonded water exhibits a broad OH stretch band at 3195 cm^–1^ with a shoulder at around 3379 cm^–1^, while destructured hydrogen-bonded water has a broad OH stretch band at about 3444 cm^–1^. Therefore, positions 3200 cm^–1^ and 3420 cm^–1^ were taken as signatures of structured and destructured hydrogen bonding, respectively, for comparison in different acid-mediated hydrogels. Figure 6a–c respectively presents the Raman spectra of [GMP-Acids], [KCl-GMP-Acids], and [KOH-GMP-Acids] hydrogels in the high wavenumber region (>2000 cm^–1^), and the Raman intensity ratios of 3200 cm^–1^ and 3420 cm^–1^ in the spectra were tabulated in Figure 6d. The lowest ratios were found in all HCl-mediated cases compared to those in PA- and H_3_PO_4_-mediated ones, implying a more disordered hydrogen bonding network. In addition, PA-mediated hydrogels had the largest ratio, compared to that of H_3_PO_4_ and HCl in the K^+^-coordinated GMP hydrogels, and the tendency of PA > H_3_PO_4_ > HCl was observed, indicating that PA-mediated hydrogels were likely to present a more-structured hydrogen bonding.

**Figure 6:**
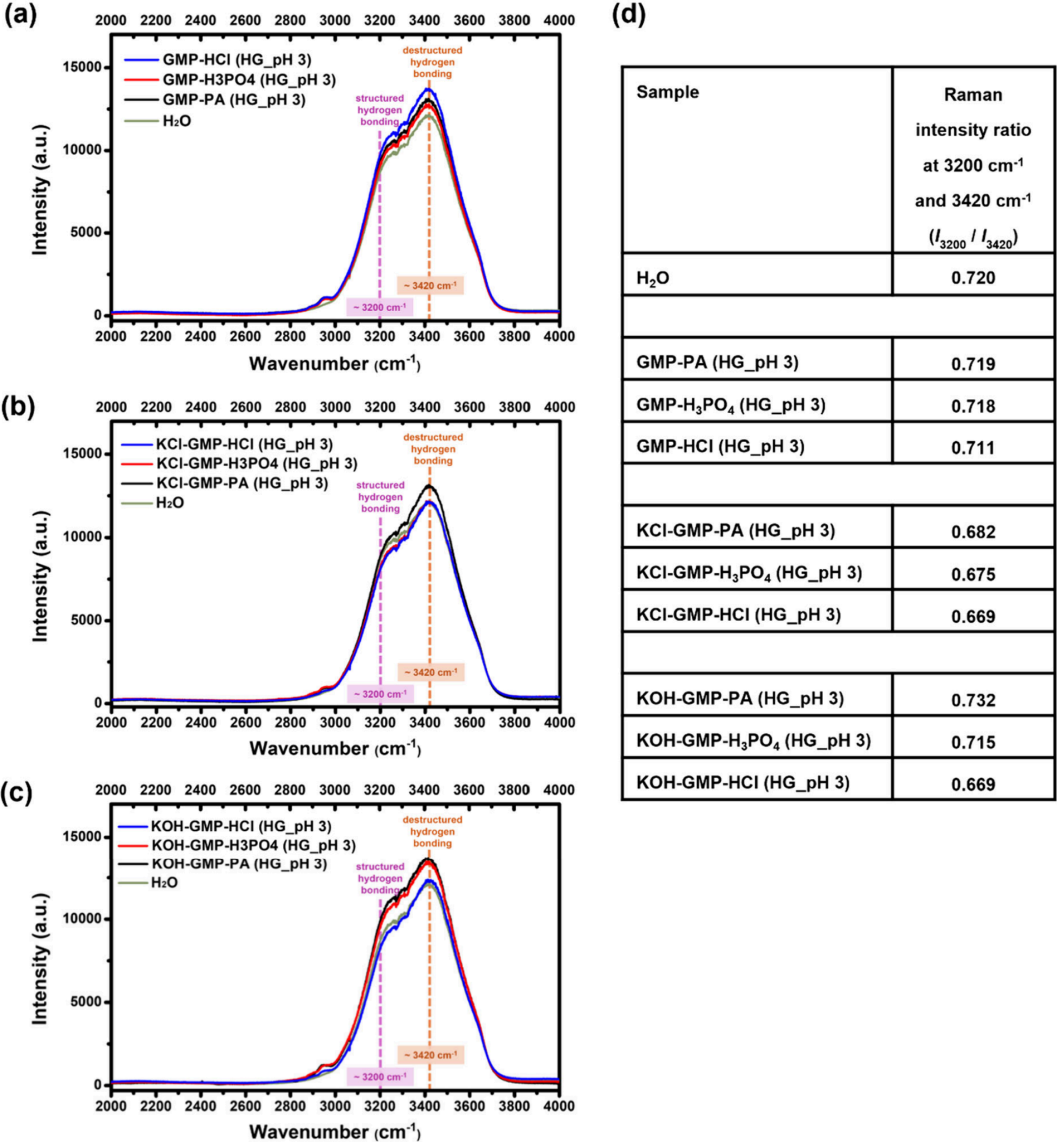
Raman spectra in the high wavenumber region (>2000 cm^–1^): (a) [GMP-Acids], (b) [KCl-GMP-Acids], and (c) [KOH-GMP-Acids] hydrogels (“Acids” referred to PA, H_3_PO_4_, or HCl). (d) Calculated Raman intensity ratios at positions 3200 cm^–1^ and 3420 cm^–1^ for respective acid-mediated hydrogels.

To further dissect the interactions of PA-mediated G-quadruplex assembly in the hydrogels, the Raman spectra were measured at different PA concentrations (20, 40, 60, and 82 mM) in the presence of fixed [GMP] = 100 mM in [KOH-GMP-PA] hydrogel system (pH 3) (before Raman measurement, tube inversion test (Figure S13­(a)) and UV–vis measurements (Figure S13­(b)) were performed to confirm the G-quadruplex and hydrogel formation at these conditions). As shown in Figure 7a, Raman measurements revealed the appearance of 860 cm^–1^ at a low PA concentration (20 mM), and the peak was gradually shifted to 850 cm^–1^ with increasing PA. PA concentration titration curves and a quantitative fitting model were applied to describe the continuous evolution of spectral shifts (Figure S14). It has been known that the pure PA solution has a characteristic peak at 856 cm^–1^ for the symmetric P–OH stretching mode of the phosphate group at pH 1.^44^ Upon adjusting the pH of the PA solution to 3 with KOH, the peak was red-shifted to 850 cm^–1^ (Figure S12). This shift could be due to the electrostatic attraction of the K^+^ with the negatively charged hydroxyl group of the phosphate in the dissociated state, which led to an increase in the bond length. As shown in eq S2 and eq S4, the vibration frequency decreased as a result of decreased force constant by longer bond length. A shift of the characteristic peak toward the lower wavenumber (856 cm^–1^ to 850 cm^–1^) was thus observed.

**Figure 7:**
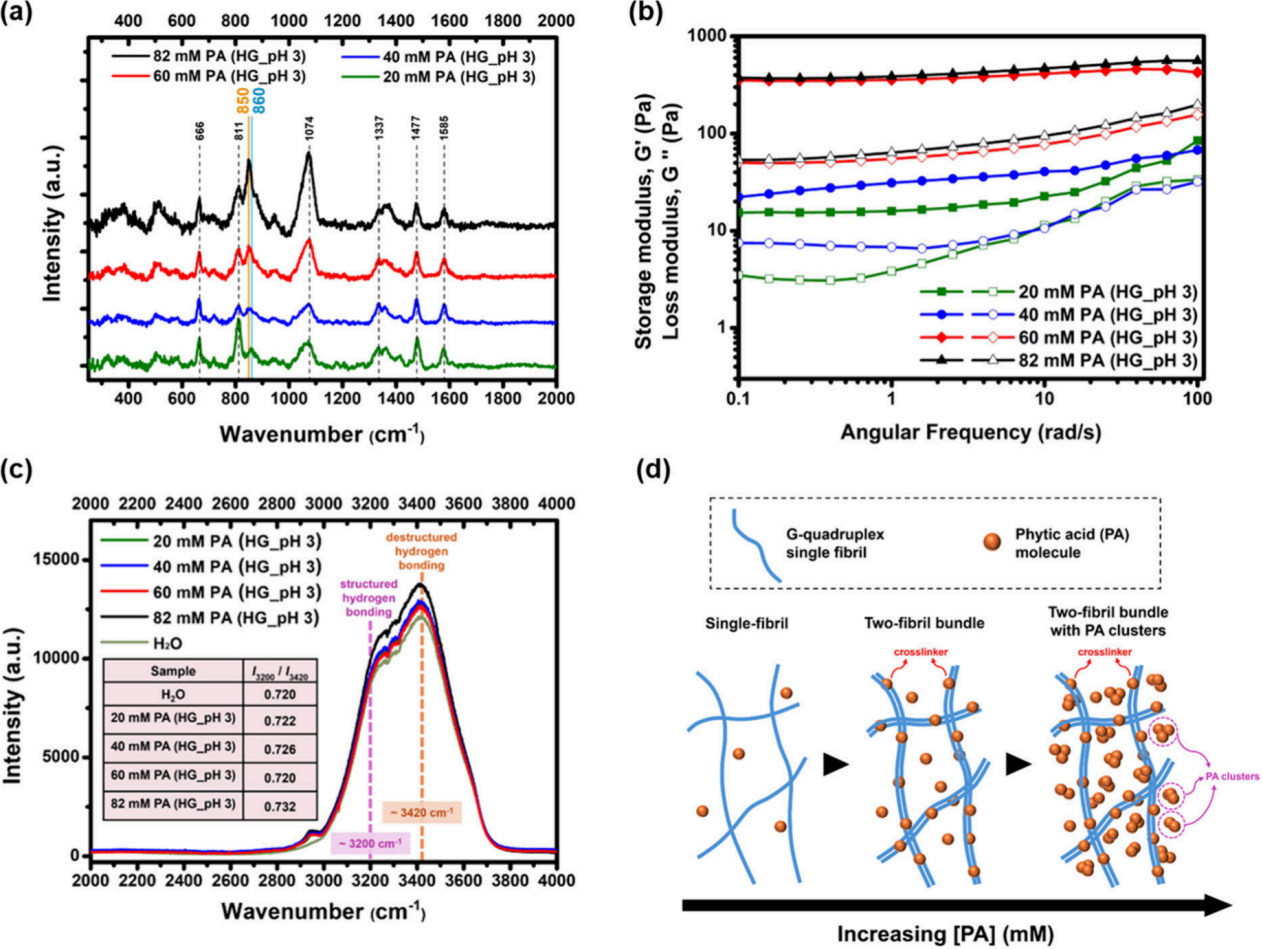
PA concentration effect on [KOH-GMP-PA] hydrogel (HG) formation (pH 3): (a) Raman peak changes between 850 and 860 cm^–1^, (b) rheological measurements and (c) Raman spectra in the high wavenumber region (>2000 cm^–1^) (inset: calculated Raman intensity ratios at 3200 cm^–1^ and 3420 cm^–1^ for respective hydrogel samples). (d) The proposed mechanism of PA-mediated G-quadruplex assembly of hydrogel.

We suspected that the peak shift of 860 cm^–1^ to 850 cm^–1^ with increasing PA in the [KOH-GMP-PA] hydrogels referred to vibration frequency changes in the P–OH stretching mode. As also observed in Figure S8 and Figure 4, the P–OH stretching mode in the [GMP-PA] and [KCl-GMP-PA] hydrogels was at 860 cm^–1^, whereas in the [KOH-GMP-PA] hydrogel, the peak appeared at 850 cm^–1^. At low PA concentration, most PA molecules served as the cross-linker between G-quadruplex fibrils. PA could interact with the G-quadruplex fibrils to make bundles via hydrogen bonding formation with GMP phosphate or hydroxyl group of sugars, leading to the Raman band appearing at a relatively high wavenumber (860 cm^–1^), because of the lower dissociation state. Alternatively, at high PA, although there were still some PA acts as the cross-linker, the excess PA exhibits a high dissociation degree, and the Coulomb’s force increases the bond length and decreases the bond order, resulting in a low wavenumber shift of the Raman band (850 cm^–1^). Since the concentration of PA in [GMP-PA] and [KCl-GMP-PA] hydrogels was about 14–17 mM (Figure S15­(a) and S15­(b)), the relatively low concentration resulted in a low dissociation level, and, thus, the Raman band at high wavenumber (860 cm^–1^) was expected (Figure S8­(a) and S8­(b)). This idea is supported by the MCR-ALS analysis of the concentration dependence that the PA band at 860 cm^–1^ (and the hydrogen-bonded form at 811 cm^–1^) predominates below 20 mM and shifts to 850 cm^–1^ above 30 mM (Figure S16).

Monitoring the changes of Raman shift between 860 cm^–1^ and 850 cm^–1^ theoretically could serve as an indicator to estimate the amount of PA that interacted with the G-quadruplex fibrils. A doublet peak at 850 cm^–1^ and 860 cm^–1^ was observed in the presence of 40 mM PA and increasing PA to 60 mM only peak at 850 cm^–1^ was found. The critical concentration of PA to interact with G-quadruplex fibrils was likely within this range to form stable hydrogels. The rheological measurements somehow reinforced this finding. The mechanical strength of the hydrogel was increased with increasing PA (Figure 7b) and a remarkable increase (>1 order) was especially found when increasing [PA] from 40 mM to 60 mM. It has been known in our SAXS measurement that free PA may aggregate to form clusters in the hydrogel (Figure S5). At [PA] = 60 mM, the extra PA molecules that are not bound with G-quadruplex fibrils may form clusters and be present in the hydrogel void, improving the mechanical strength of hydrogel. Exploring the PA concentration effect on the water molecules in [KOH-GMP-PA] hydrogels, the Raman spectra in the high wavenumber region (>2000 cm^–1^) revealed there was not much difference at [PA] < 60 mM but a more structured hydrogen bonding was observed with increasing PA at 82 mM (Figure 7c). The excess PA molecules that form clusters potentially restructure the hydrogen bonding network of the water molecules in the hydrogel. The proposed PA-mediated G-quadruplex assembly of hydrogel is illustrated in Figure 7d. While the current study elucidated the PA-mediated assembly mechanism, further investigation of water dynamics represents a valuable direction for future research. Understanding how PA specifically influences the hydrogen bonding networks in these hydrogels provides deeper mechanistic insights into the assembly process and the resulting material properties.

## Experimental Section

### Preparation of Acid-Mediated Guanosine-5′-Monophosphate Hydrogels

The GMP and KCl stock solutions were prepared at 1 and 1.459 M, respectively, in ddH_2_O. The KOH solution (45% w/w) was diluted with ddH_2_O to become 1.4592 M. HCl, H_3_PO_4_, and PA aqueous solutions were prepared at 1 M by ddH_2_O. For PA-mediated GMP self-assembly, potassium ion (K^+^ sources either from KCl_(aq)_ or KOH_(aq)_), GMP, and PA were added sequentially, with concentrations adjusted to the desired concentrations (Supporting Information and Methods; Table S3–S5) to investigate their respective concentration effects on gelation. Different acid (HCl, H_3_PO_4_, or PA)-mediated GMP assembly was also accordingly examined at pH 3.

### UV-vis Spectroscopy

UV–vis absorption spectra were obtained by a UV-vis spectrophotometer (Model V-660, Jasco, Japan). The samples were placed on a CaF_2_ window and covered with another piece with a path length of 0.01 mm. The spectra were acquired in the range 200–450 nm with a scanning speed of 100 nm/min. The bandwidth of the instrument was set to 1.0 nm, the data interval was 0.2 nm, and for each measurement, the average absorbance value of three scans was used for analysis. The turbidity of [KCl-GMP-PA] and [KOH-GMP-PA] hydrogels was assessed at *A*_600_.

### Raman Spectroscopy (532 nm Confocal Raman Microspectrometer Setup)

Detailed instrumental setup is shown in the Supporting Information (Figure S17). The signal accumulation of each single Raman spectrum was 40 s, repeated 10 times at 532 nm laser excitation (average power = 40 mW). Regarding the data processing, the low wavenumber region (<250 cm^–1^) and fingerprint region (250–2000 cm^–1^) Raman spectra of all samples were first selected for intensity normalization at 1640 cm^–1^ (HOH bending of solvent H_2_O), and the solvent H_2_O was subtracted after normalization. Next, after masking the range of feature peaks by IGOR Pro (v7.08) software, the baseline was fitted by polynomial and then subtracted. High wavenumber (>2000 cm^–1^) Raman measurements were plotted as raw data without any normalization and baseline correction. Calibration of the Raman shift was done by measuring the standard atomic emission spectra (Ne lamps).

### SAXS/WAXS Analysis

The samples were loaded into the cavities of the liquid cell (path length = 2.5 mm) and sealed with polyimide kapton and O-rings. The Taiwan Photon Source (TPS) at beamline 13A, National Synchrotron Radiation Research Center (NSRRC), Taiwan, was employed for SAXS/WAXS measurements. The photon energy was 15 keV. The photon energy is 15 keV, and the distance between the sample and detector is 2285.3 mm.

### Rheological Tests

Rheological tests of synthetic hydrogels (1 mL) were carried out on the MCR 302e rheometer (Anton Paar, Austria) using a sandblasted parallel plate geometry (PP25/S) with a diameter of 25 mm, in conjunction with a Peltier temperature control device (P-PTD200). (The temperature was controlled at 25 °C.) Frequency sweep experiments were performed in the range of 0.1 to 100 rad/s with a constant strain of 0.2% to determine the storage modulus (*G*′) and loss modulus (*G*″) of the hydrogels. The loss factor tan δ = *G*”/*G*′ was calculated, and the rheological fitting was conducted (see Figure S4 and Tables S1 and S2 in the Supporting Information).

## Conclusion

In this study, the PA-mediated, GMP self-assembled hydrogels were explored by Raman spectroscopy along with SAXS and rheological measurements. A newly identified Raman peak in the low wavenumber region (∼96–110 cm^–1^) can serve as a unique marker of G-tetrad packing and is cation-specific. The hydrogels made from different K^+^ sources (i.e., [KCl-GMP-PA] and [KOH-GMP-PA]) were of varying gel textures, which could be attributed to the distinct pH in the two systems. In the presence of equal amounts of K^+^, GMP, and PA, multiple fibrils tended to assemble into bundles in the relatively acidic hydrogel [KCl-GMP-PA] than in the [KOH-GMP-PA], resulting in increasing gel mechanical strength as well as turbidity. Alternatively, by keeping pH consistent at 3, the PA concentration was critical in maintaining the gel matrix and the resulting mechanical strength. Monitoring the P–OH stretching mode (850–860 cm^–1^) and water-associated hydrogen bonding network at the high wavenumber region (>2000 cm^–1^) of Raman spectra indicated that at low [PA], the PA molecules acted as the cross-linkers of G-quadruplex fibrils in the hydrogel. As [PA] increased, the excess PA was likely to aggregate, forming clusters in the hydrogel void to support the gel matrix, further improving the mechanical strength. Our multiregion Raman analysis strategy combines low-frequency (lattice modes), fingerprint (molecular vibrations), and high-frequency (water structure) regions to provide a comprehensive picture of hydrogel organization from molecular to network scales. This approach reveals previously inaccessible information about cross-linker-fibril interactions and water structuring in the native gel state without the need for any sample pretreatment. By monitoring the low wavenumber region (∼96–110 cm^–1^) and the established quantitative correlation in PA concentration-dependent Raman peak shifts (850 ↔ 860 cm^–1^), these Raman markers potentially could be applied to in-line quality controls of GMP-based hydrogel synthesis to ensure the desirable gel textures and cation identity, providing a new analytical framework for rational hydrogel design.

## Supplementary Material



## Notes

Additional experimental details and methods; rheological data; photographic images of tube inversion tests; UV–vis spectra, Raman spectra, and additional control experiments and data analysis; ChemDraw 3D-constructed molecular model (PDF)
