# Supplementary material for: In-Situ Exploration of Phytic Acid-Mediated Supramolecular Self-Assembly and Gelation
Source: Anal Chem. 2026 Jan 30;98(5):3711–22. doi: 10.1021/acs.analchem.5c05630 (PMC12903057; doi:10.1021/acs.analchem.5c05630)
Supplement: Supplementary file 1 [file ac5c05630_si_001.pdf]

# Supporting Information

## In-situ exploration of phytic acid-mediated supramolecular self-assembly and gelation

Yu-Sheng Yen[1,#], Chia-Wei Zhang[1,#],Wei-Tsung Chuang[2], Chun-Fu Chang[1,*], Hirotsugu Hiramatsu[1,*] and Hsin-Yun Hsu[1,3,*]

[1]Department of Applied Chemistry and Institute of Molecular Science, National Yang-Ming Chiao-Tung University, Hsinchu 300093, Taiwan.

[2]National Synchrotron Radiation Research Center (NSRRC), Hsinchu 300092, Taiwan

[3]Center for Emergent Functional Matter Science, National Yang-Ming Chiao-Tung University, Hsinchu 300093, Taiwan.

[#] Y.-S. Yen and C.-W. Zhang contributed equally to this work.

*Corresponding authors:
hyhsu99@nycu.edu.tw (H.-Y. Hsu)
Department of Applied Chemistry and Institute of Molecular Science, National Yang-Ming Chiao-Yung University, No.1001 Ta-Hsueh Road, Hsinchu 300093, Taiwan
Center for Emergent Functional Matter Science, National Yang-Ming Chiao-Tung University, No.1001 Ta-Hsueh Road, Hsinchu 300093, Taiwan.

hiramatu@nycu.edu.tw (H. Hiramatsu) and chunfuc@nycu.edu.tw (C.-F. Chang)
Department of Applied Chemistry and Institute of Molecular Science, National Yang-Ming Chiao-Yung University, No.1001 Ta-Hsueh Road, Hsinchu 300093, Taiwan

## Table of Contents


**Supplementary Data**

**Supplementary Tables**

Table S1. Fitting G' and G" with frequency data in [KCl-GMP-PA] hydrogels S3

Table S2. Fitting G' and G" with frequency data in [KOH-GMP-PA] hydrogels S4

**Supplementary Figures**

Figure S1. GMP, PA, and KCl concentration effects on [KCl-GMP-PA] hydrogel formation S5

Figure S2. Turbidity of [KCl-GMP-PA] and [KOH-GMP-PA] hydrogel S6

Figure S3. PA and KOH concentration effects on [KOH-GMP-PA] hydrogel formation S6

Figure S4. Loss factor curves of [KCl-GMP-PA] and [KOH-GMP-PA] hydrogels S7

Figure S5. G-tetrad molecular model and two-fibril bundle schematic S8

Figure S6. Rheological measurements of [GMP-Acids], [KCl-GMP-Acids], and [KOH-GMP-Acids] hydrogels S9

Figure S7. UV-Vis spectra of [GMP-Acids], [KCl-GMP-Acids], and [KOH-GMP-Acids] hydrogels S10

Figure S8. Raman spectra of [GMP-Acids], [KCl-GMP-Acids], and [KOH-GMP-Acids] hydrogels S11

Figure S9. Low wavenumber region (<250 $cm^{-1}$) Raman spectra under continuous irradiation, at different laser powers, and polarization-resolved measurements S12

Figure S10. Low wavenumber region (<250 $cm^{-1}$) Raman spectra at different concentrations of GMP, KCl, varied pH, and with different nucleotide monophosphates S13

Figure S11. Temperature-controlled structural reversibility of G-tetrad assembly S14

Figure S12. Raman spectra of PA, $H_3PO_4$, and HCl solutions S15

Figure S13. PA concentration effect on [KOH-GMP-PA] hydrogel formation S15

Figure S14. Calibration curve of Raman peak shifts versus PA concentration S16

Figure S15. Photographic images of tube inversion test S17

Figure S16. Singular value decomposition (SVD) and MCR-ALS analysis S18

**Supplementary Materials and Methods**

Chemicals S21

Preparation of acid-mediated GMP hydrogels (Table S3-S5) S21

Raman spectroscopy setup (Figure S17) S22

Rheological fitting S24

**References** S26

# Supplementary Data

## Supplementary Tables

**Table S1.** Fitting G’ and G” with frequency data in [KCl-GMP-PA] hydrogels: (a) [GMP], (b) [PA], and (c) [$K^+$] concentration effects.

**(a) G’**

| **[GMP] (mM)** | **120** | **100** | **80** |
|---|---|---|---|
| **C** | 37469.38 | 23049.31 | 12098.72 |
| ***β*** | 0.09 | 0.09 | 0.02 |
| **R-Square** | 0.97 | 0.96 | 0.56 |

**G”**

| **[GMP] (mM)** | **120** | **100** | **80** |
|---|---|---|---|
| **C** | 77864.29 | 40685.06 | N.A. |
| ***β*** | 0.08 | 0.09 | |
| **R-Square** | 0.83 | 0.88 | |

**(b) G’**

| **[PA] (mM)** | **90** | **75** | **60** | **45** | **30** | **15** | **5** |
|---|---|---|---|---|---|---|---|
| **C** | 15105.06 | 17777.07 | 23049.31 | 19627.36 | 4091.14 | N.A. | 5.40 |
| ***β*** | 0.05 | 0.06 | 0.09 | 0.06 | 0.03 | | 0.78 |
| **R-Square** | 0.8 | 0.97 | 0.96 | 0.93 | 0.66 | | 0.94 |

**G”**

| **[PA] (mM)** | **90** | **75** | **60** | **45** | **30** | **15** | **5** |
|---|---|---|---|---|---|---|---|
| **C** | 34499.68 | 78325.43 | 40685.06 | N.A. | N.A. | 125.35 | 14.9 |
| ***β*** | 0.06 | 0.03 | 0.09 | | | 0.12 | 0.17 |
| **R-Square** | 0.66 | 0.55 | 0.88 | | | 0.41 | 0.95 |

**(c) G’**

| **[$K^+$] (mM)** | **500** | **400** | **364.8** | **300** | **200** | **100** |
|---|---|---|---|---|---|---|
| **C** | 27873.35 | 243730.21 | 23049.31 | 16646.55 | 5959.06 | 1364.90 |
| ***β*** | 0.09 | 0.10 | 0.09 | 0.07 | 0.08 | 0.04 |
| **R-Square** | 0.98 | 0.99 | 0.96 | 0.87 | 0.88 | 0.38 |

**G”**

| **[$K^+$] (mM)** | **500** | **400** | **364.8** | **300** | **200** | **100** |
|---|---|---|---|---|---|---|
| **C** | 89966.33 | 71149.16 | 40685.06 | 28088.52 | 7584.02 | 2909.04 |
| ***β*** | 0.05 | 0.06 | 0.09 | 0.09 | 0.13 | 0.08 |
| **R-Square** | 0.60 | 0.79 | 0.88 | 0.52 | 0.87 | 0.45 |

**Table S2.** Fitting G' and G" with frequency data in [KOH-GMP-PA] hydrogels: (a) [GMP], (b) [PA], and (c) [$K^+$] concentration effects.

**(a) G'**

| **[GMP] (mM)** | **120** | **100** | **80** | **60** | **40** | **20** |
|---|---|---|---|---|---|---|
| **C** | 16.17 | 35.66 | 13.48 | 109.74 | 275.02 | 197.72 |
| ***β*** | 0.3 | 0.28 | 0.3 | 0.08 | 0.99 | 0.99 |
| **R-Square** | 0.96 | 0.97 | 0.88 | 0.63 | 0.94 | 0.95 |

**G"**

| **[GMP] (mM)** | **120** | **100** | **80** | **60** | **40** | **20** |
|---|---|---|---|---|---|---|
| **C** | 12.31 | 62.58 | 4.66 | 22.3 | 4.42 | 3.87 |
| ***β*** | 0.33 | 0.22 | 0.51 | 0.37 | 0.62 | 0.33 |
| **R-Square** | 0.88 | 0.9 | 0.94 | 0.95 | 0.98 | 0.92 |

**(b) G'**

| **[PA] (mM)** | **90** | **75** | **60** |
|---|---|---|---|
| **C** | 1732.73 | 374.54 | 109.74 |
| ***β*** | 0.05 | 0.02 | 0.08 |
| **R-Square** | 0.91 | 0.79 | 0.63 |

**G"**

| **[PA] (mM)** | **90** | **75** | **60** |
|---|---|---|---|
| **C** | 1569.17 | 197.64 | 22.3 |
| ***β*** | 0.12 | 0.16 | 0.37 |
| **R-Square** | 0.97 | 0.81 | 0.95 |

**(c) G'**

| **[$K^+$] (mM)** | **400** | **364.8** | **300** | **200** |
|---|---|---|---|---|
| **C** | 225.5 | 109.74 | 220.06 | N.A. |
| ***β*** | 0.99 | 0.08 | 0.03 | |
| **R-Square** | 0.94 | 0.63 | 0.32 | |

**G"**

| **[$K^+$] (mM)** | **400** | **364.8** | **300** | **200** |
|---|---|---|---|---|
| **C** | 5.45 | 22.3 | 56.95 | 135.36 |
| ***β*** | 0.48 | 0.37 | 0.31 | 0.25 |
| **R-Square** | 0.93 | 0.95 | 0.85 | 0.75 |

N.A.: not available as fitting fail when $\beta$ approaches 0

## Supplementary Figures

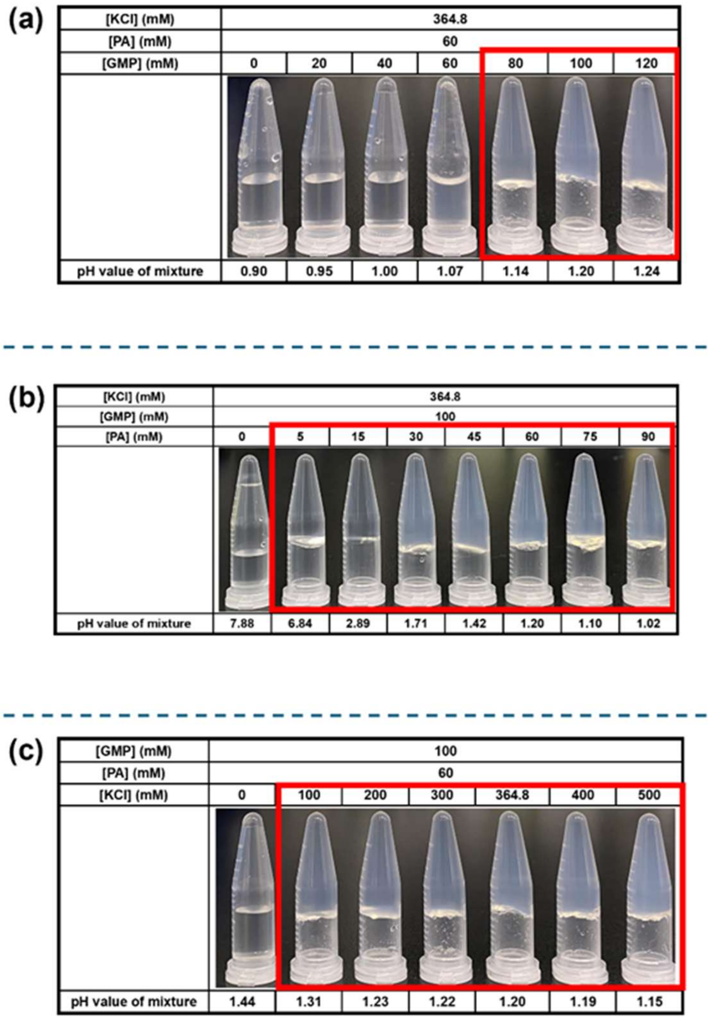


**Figure S1.** (a) GMP, (b) PA, and (c) KCl concentration effects on [KCl-GMP-PA] hydrogel formation: photographic images of tube inversion test (left; red square indicated the gel formation) and UV-Vis spectra normalized at 252 nm (right)

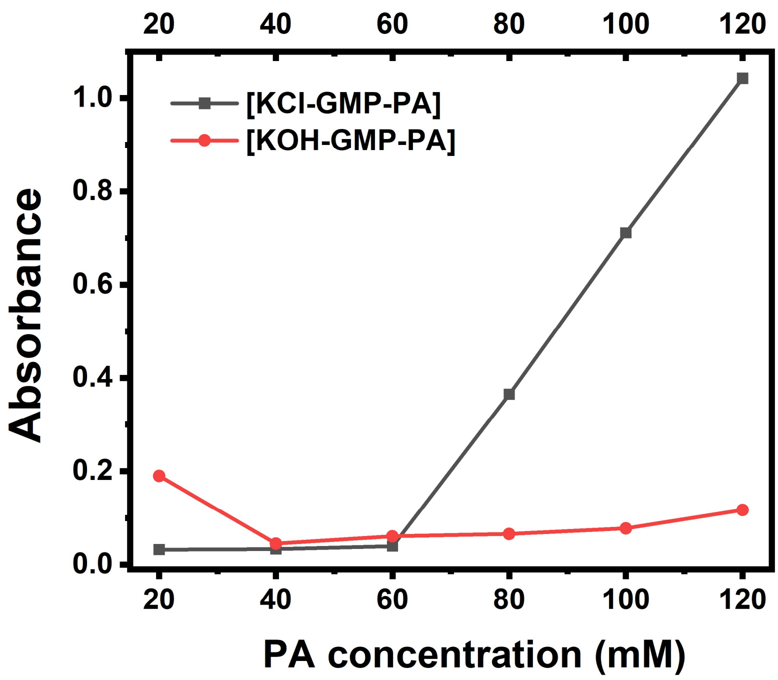


**Figure S2.** Turbidity of [KCl-GMP-PA] and [KOH-GMP-PA] hydrogel assessed at $A_{600}$

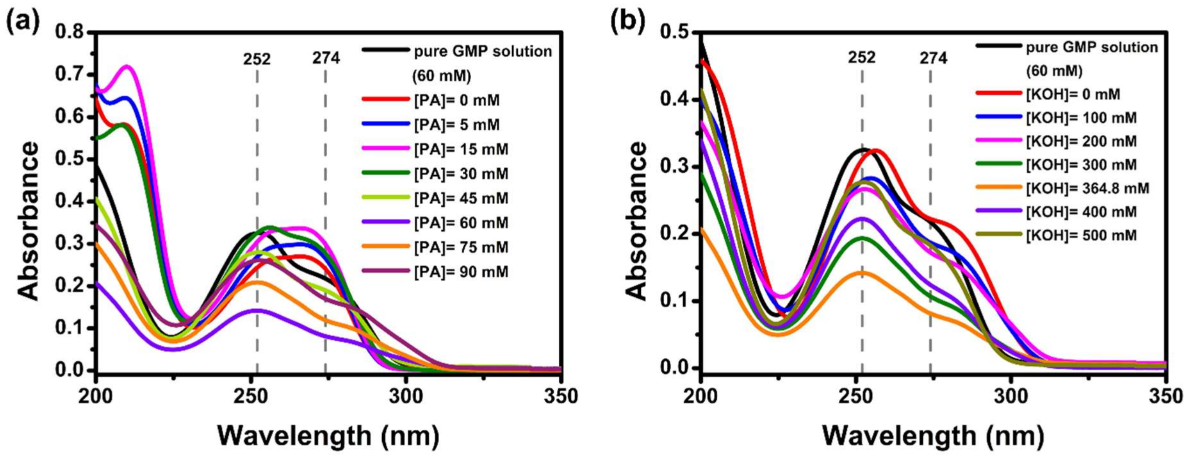


**Figure S3.** (a) PA and (b) KOH concentration effects on [KOH-GMP-PA] hydrogel formation: the UV-Vis spectra

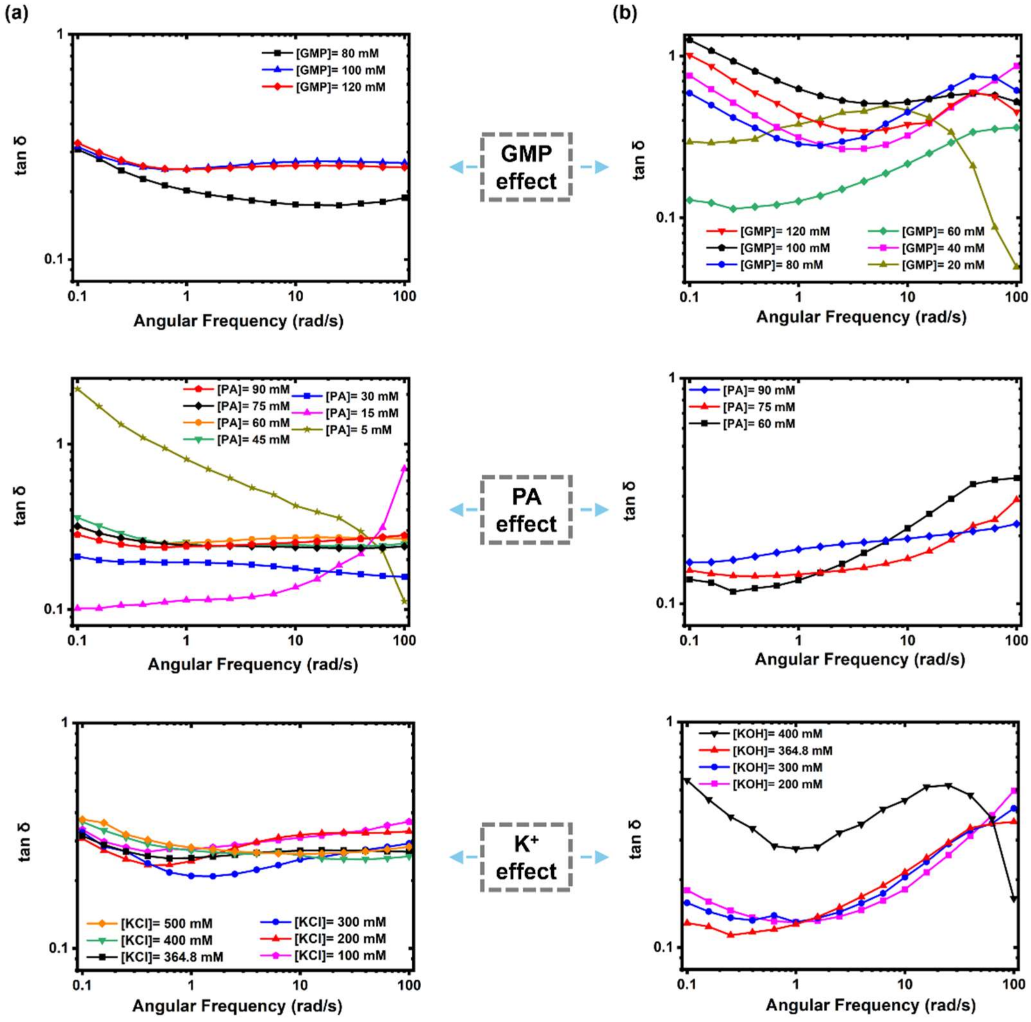


**Figure S4**. Loss factor curves of the tan δ= G"/G' of (a) [KCl-GMP-PA] and (b) [KOH-GMP-PA] hydrogels ([GMP], [PA] and [$K^+$] concentration effects were respectively compared (top, middle, and bottom))

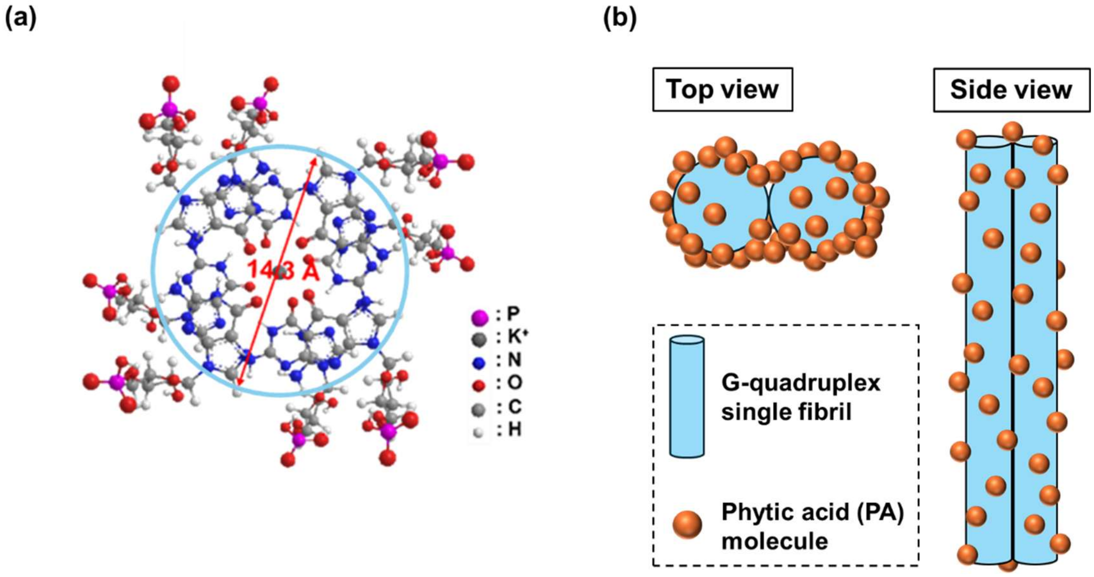


**Figure S5.** (a) A 2-layered G-tetrad molecular model was constructed using Chemdraw 3D, and was optimized by MM2 to calculate the lowest energy conformation, representing its stable 3D structure. The outer diameter of a single G-quadruplex fibril was thus estimated to be ~14.3 Å. (b) Schematic diagram of the two-fibril bundle formed in the PA-mediated GMP-based hydrogel.

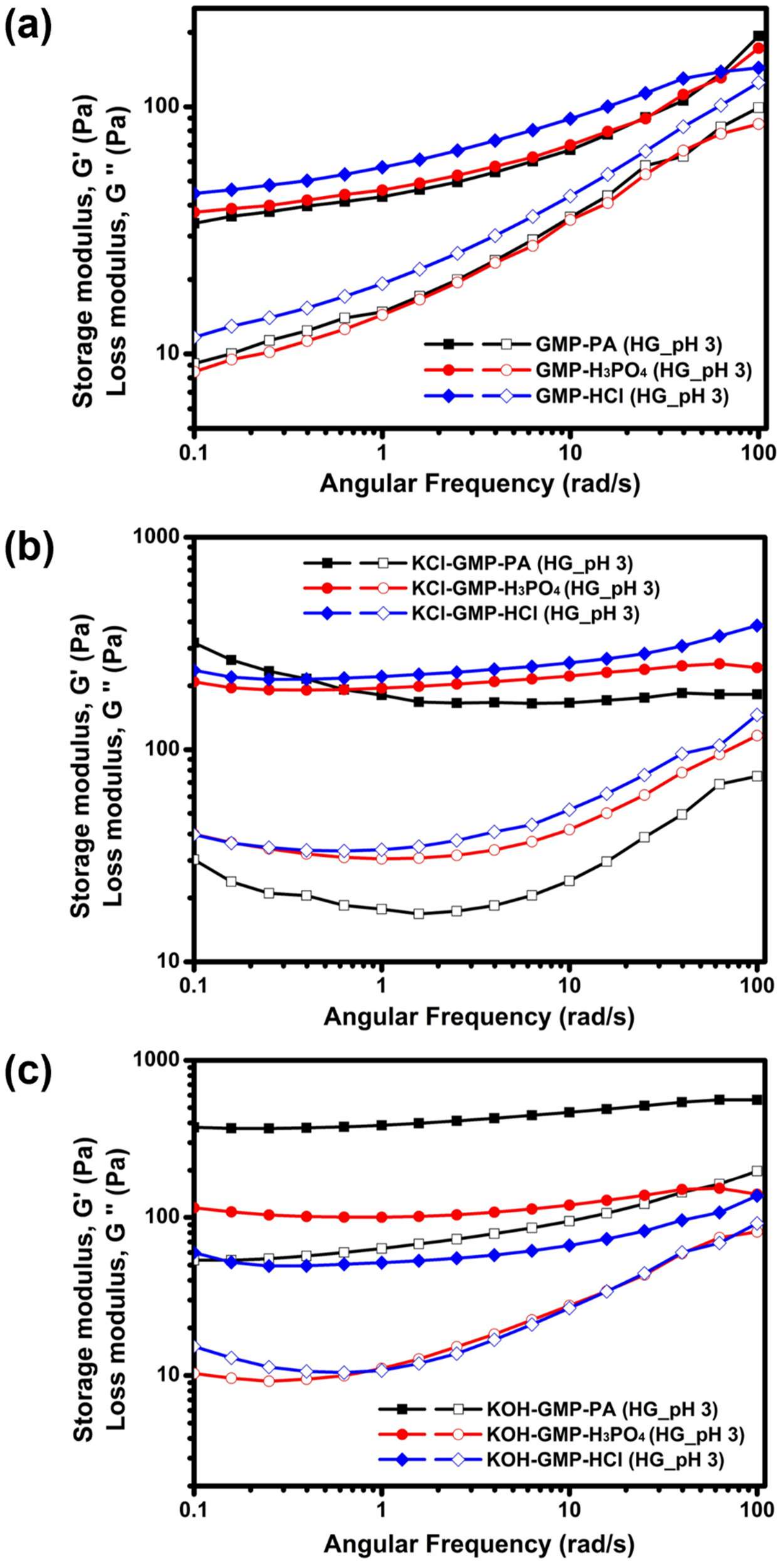


**Figure S6**. Rheological measurements of (a) [GMP-Acids], (b) [KCl-GMP-Acids], and (c) [KOH-GMP-Acids] hydrogels ("Acids" referred to PA, $H_3PO_4$, or HCl).

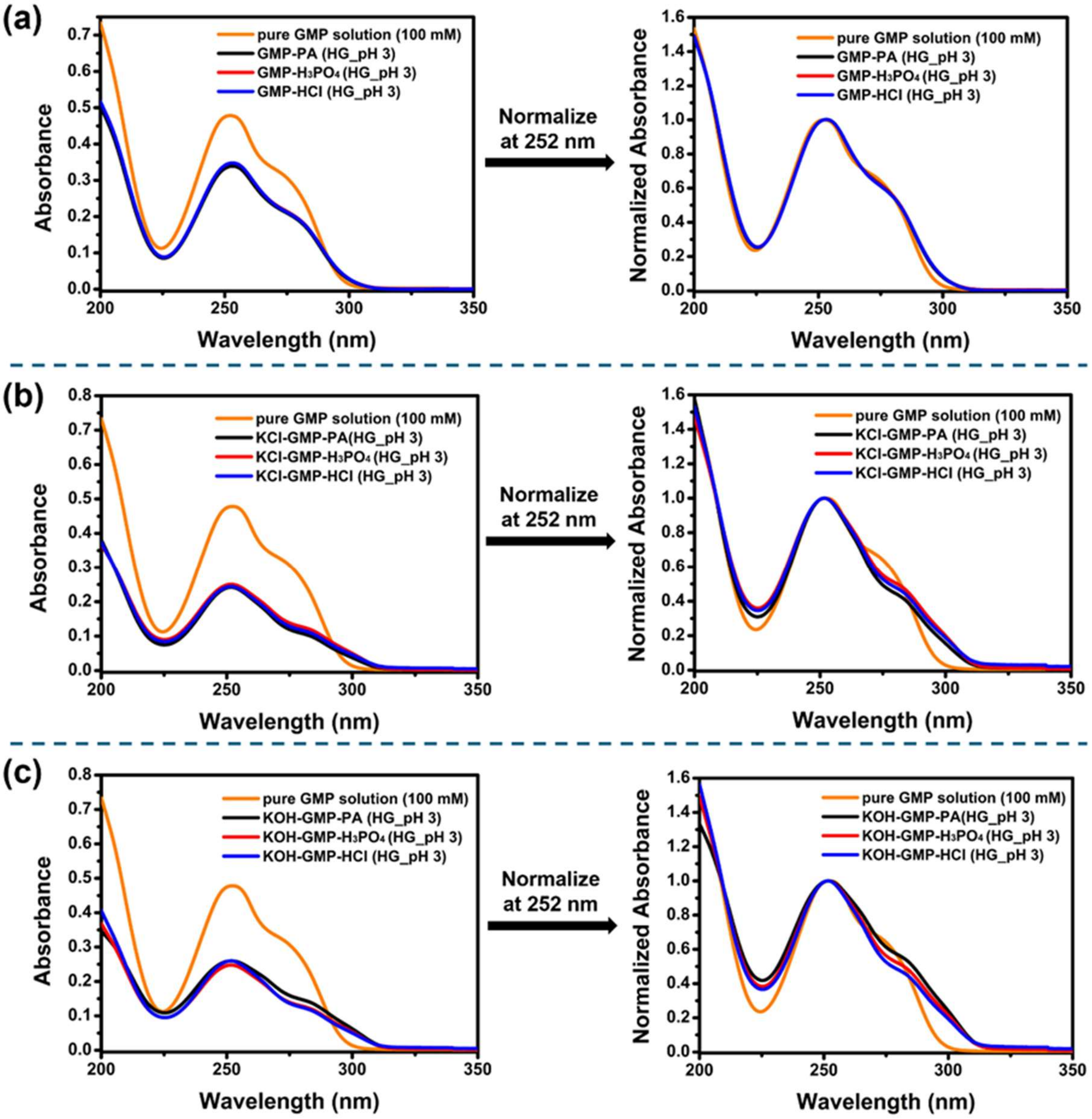


**Figure S7.** UV-Vis spectra and the spectra normalized at 252 nm: (a) [GMP-Acids], (b) [KCl-GMP-Acids], and (c) [KOH-GMP-Acids] hydrogels ("Acids" referred to PA, $H_3PO_4$, or HCl).

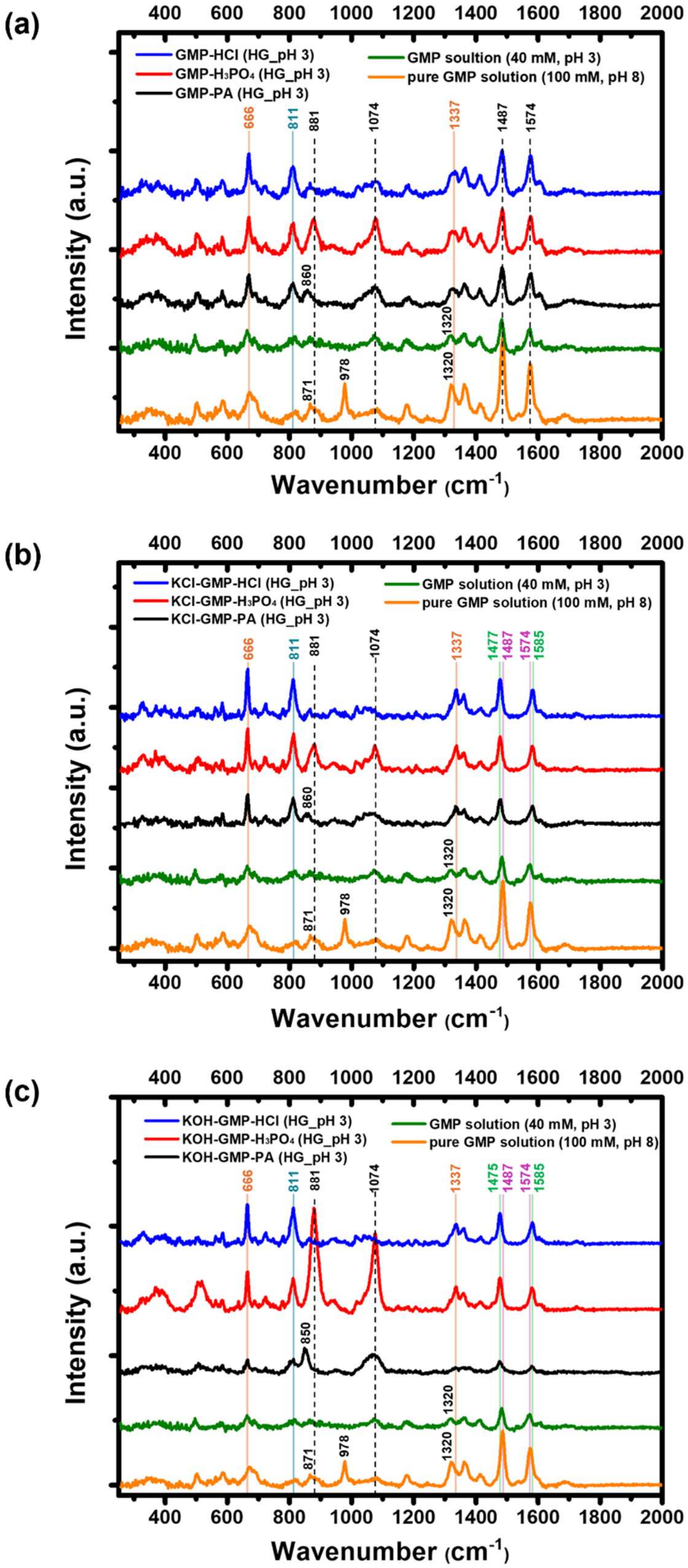


**Figure S8.** Raman spectra (raw data) of (a) [GMP-Acids], (b) [KCl-GMP-Acids], and (c) [KOH-GMP-Acids] hydrogels ("Acids" referred to PA, $H_3PO_4$, or HCl).

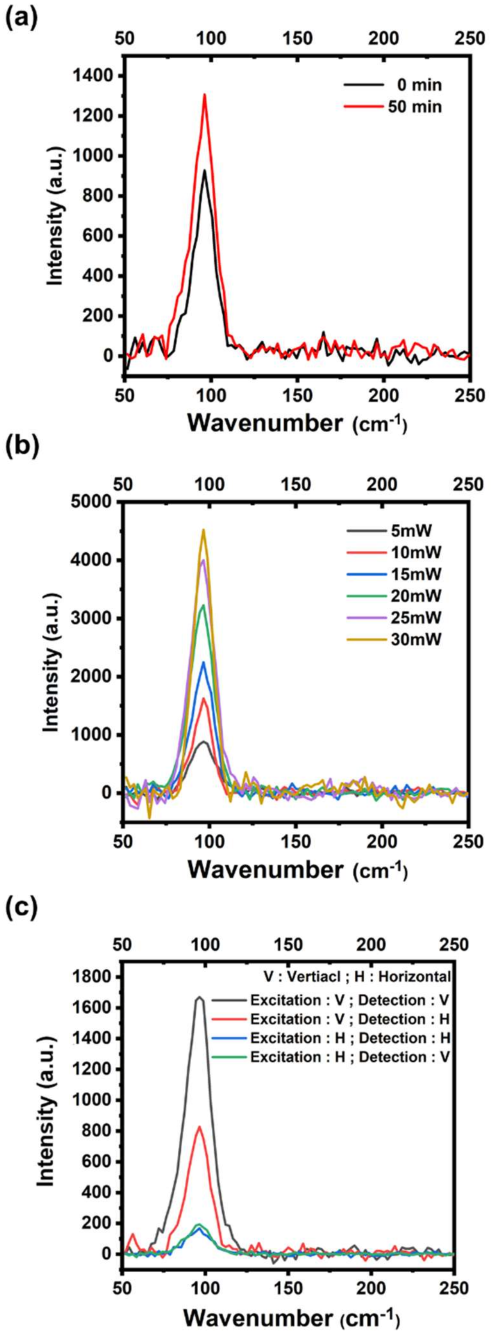


**Figure S9.** The low wavenumber (<250 cm$^{-1}$) region Raman spectra of [KCl-GMP-HCl] hydrogel sample acquired under (a) continuous irradiation at 5 mW for 50 min and (b) at different laser powers (200-seconds exposure for each spectrum). (c) Polarization-dependent Raman spectra measured at four different polarization configurations.

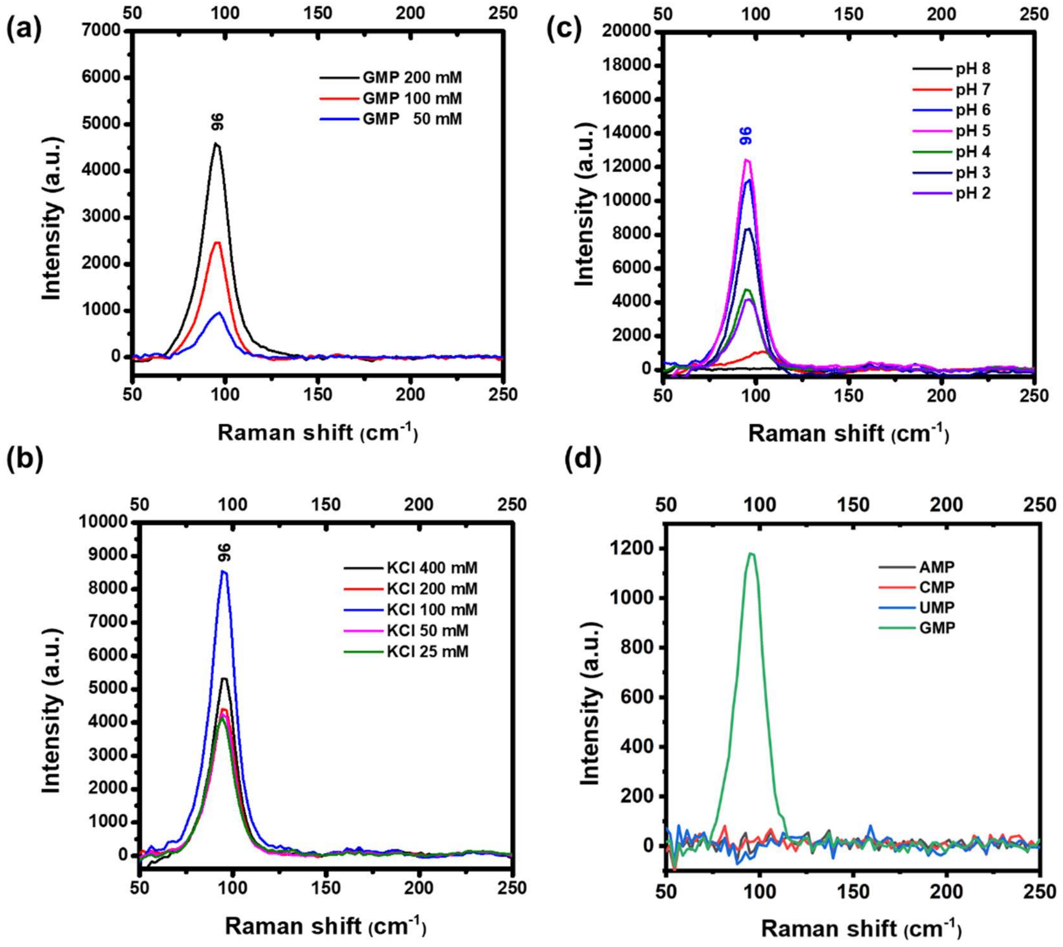


**Figure S10.** The low wavenumber (<250 cm$^{-1}$) region Raman spectra of [KCl-GMP-HCl] at different concentrations of (a) GMP, (b) KCl, and (c) at varied pH. (d) GMP was replaced with AMP, CMP, or UMP to confirm the specificity of the ~96–110 cm$^{-1}$ marker to G-tetrads

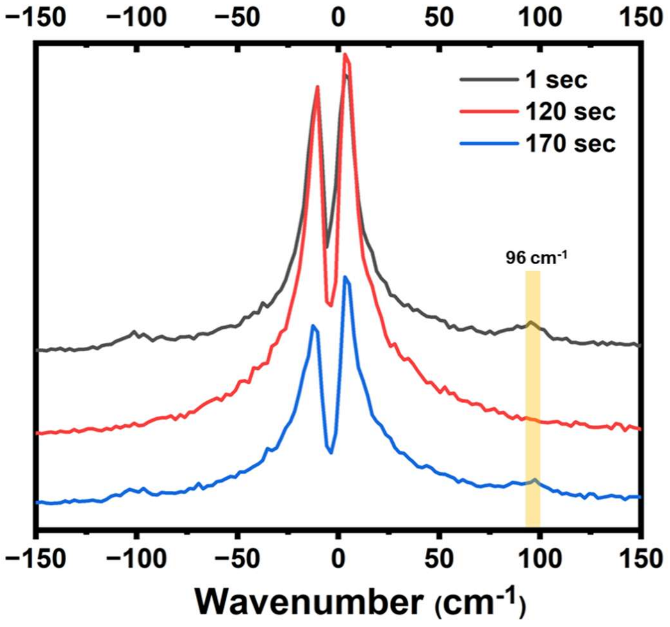


**Figure S11.** Structural reversibility of the G-tetrad assembly monitored by temperature-controlled Raman experiment. The experiment was conducted by heating the [KCl-GMP-HCl] hydrogel sample to 140 °C using a hot-air gun (FR-802, HAKKO). The characteristic Raman band at 96 $cm^{-1}$ appears in both Stokes and anti-Stokes regions, which confirms this signal is a real Raman band instead of spectral artifact. The 96-$cm^{-1}$ band disappears at approximately 120 seconds, indicating that the GMP molecules largely dissociate into their monomeric form at high temperature. The heat source was then turned off. The 96 $cm^{-1}$-band reappears upon cooling at around 170 seconds, demonstrating the reassembly of GMP molecules to form the G-tetrad and eventually the fibril structures.

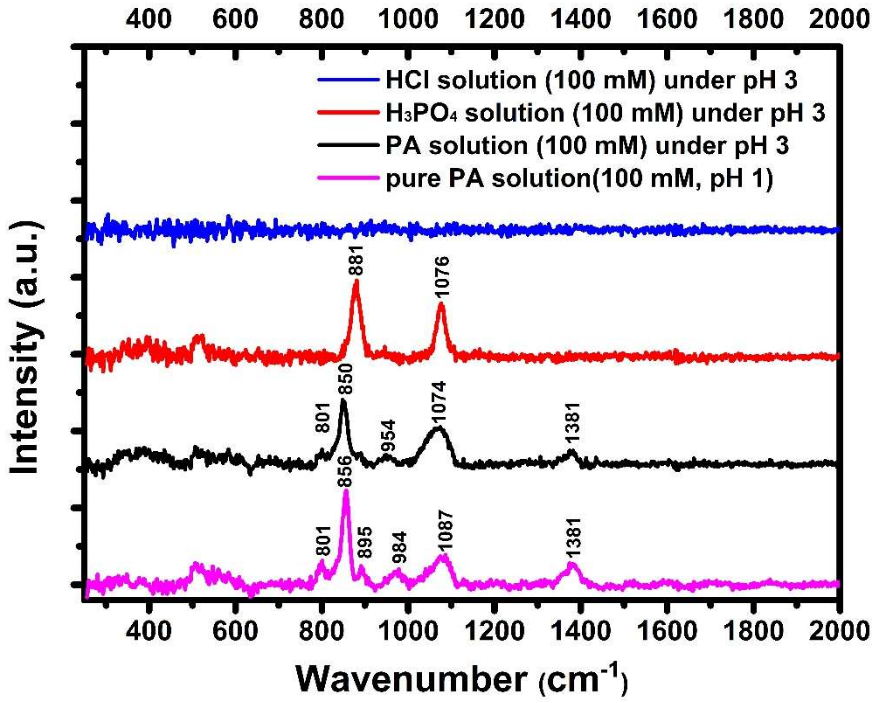


**Figure S12.** Raman spectra of pure PA solution (pH 1) and PA, $H_3PO_4$, and HCl solution adjusted to pH 3 by KOH.

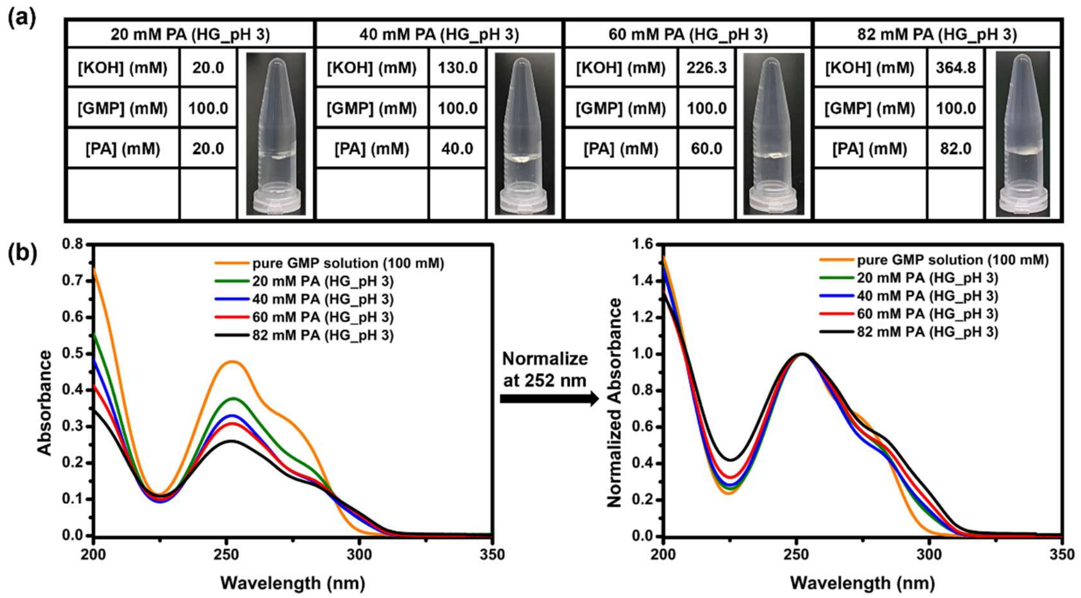


**Figure S13.** PA concentration effect on [KOH-GMP-PA] hydrogel (HG) formation (at pH 3): (a) photographic images of tube inversion test, (b) UV-Vis spectra and the spectra normalized at 252 nm.

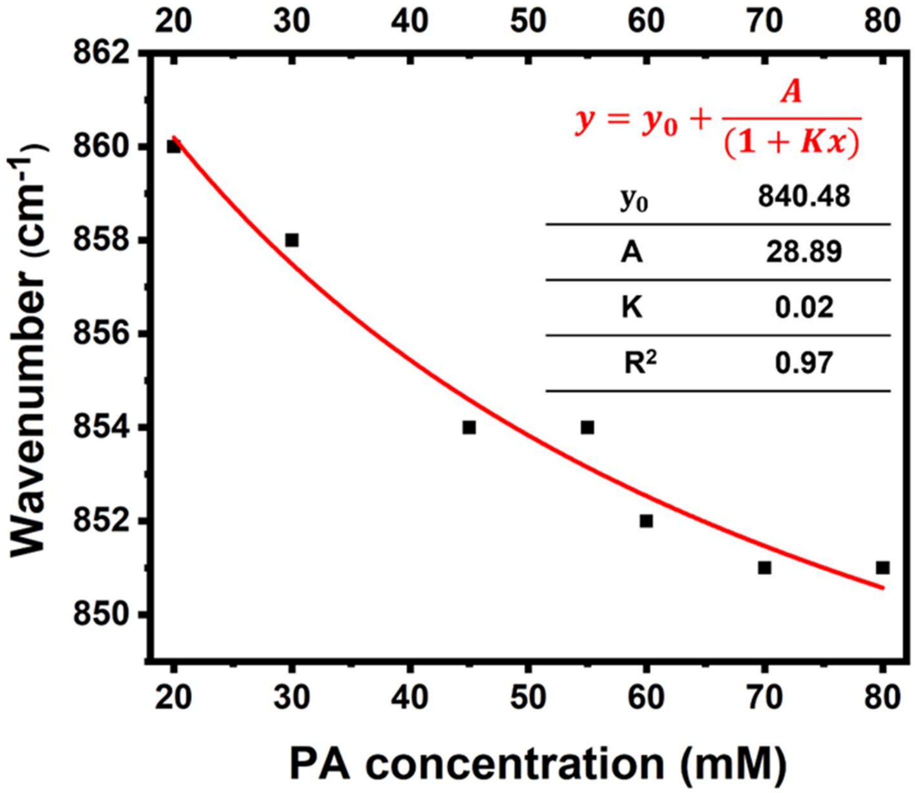


**Figure S14.** The calibration curve of Raman peak shifts (850↔860 cm$^{-1}$) versus [PA] concentration in [KOH-GMP-PA] (pH 3) hydrogel, and a quantitative fitting model was employed.

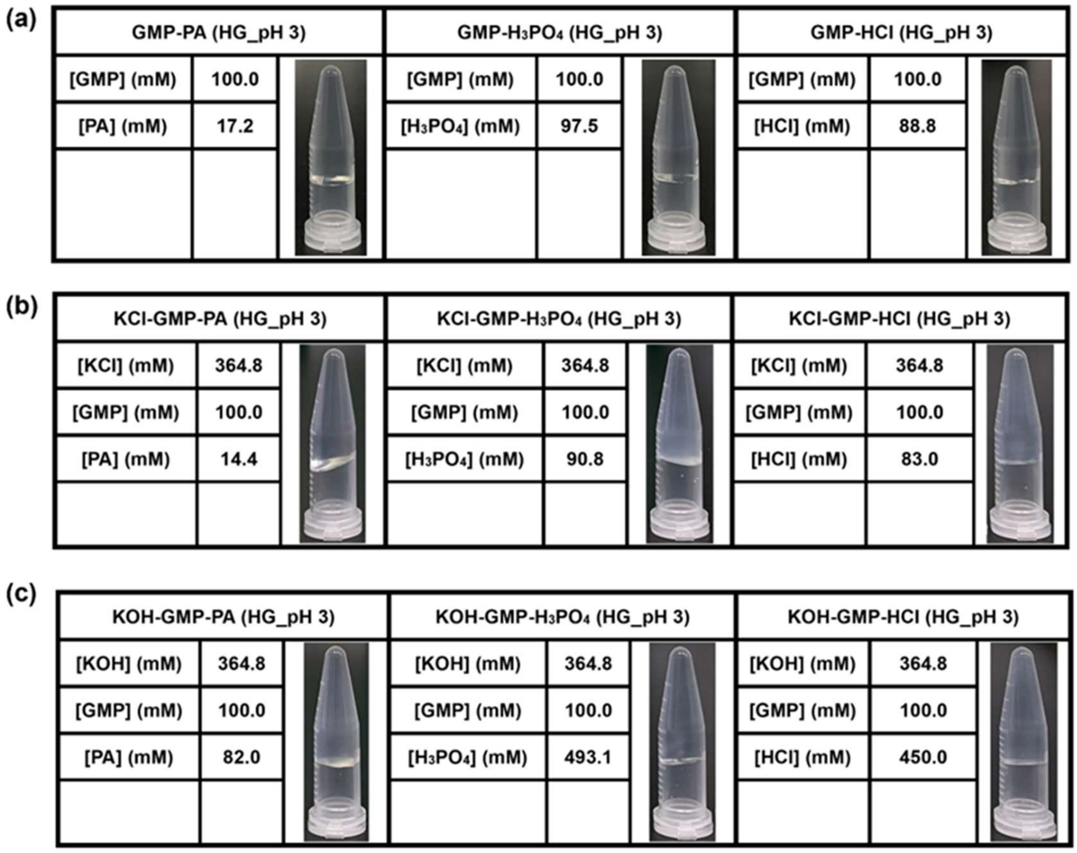


**Figure S15.** Photographic images of tube inversion test: (a) [GMP-Acids], (b) [KCl-GMP-Acids], and (c) [KOH-GMP-Acids] hydrogels (in (a), (b), and (c), "Acids" referred to PA, $H_3PO_4$, or HCl)

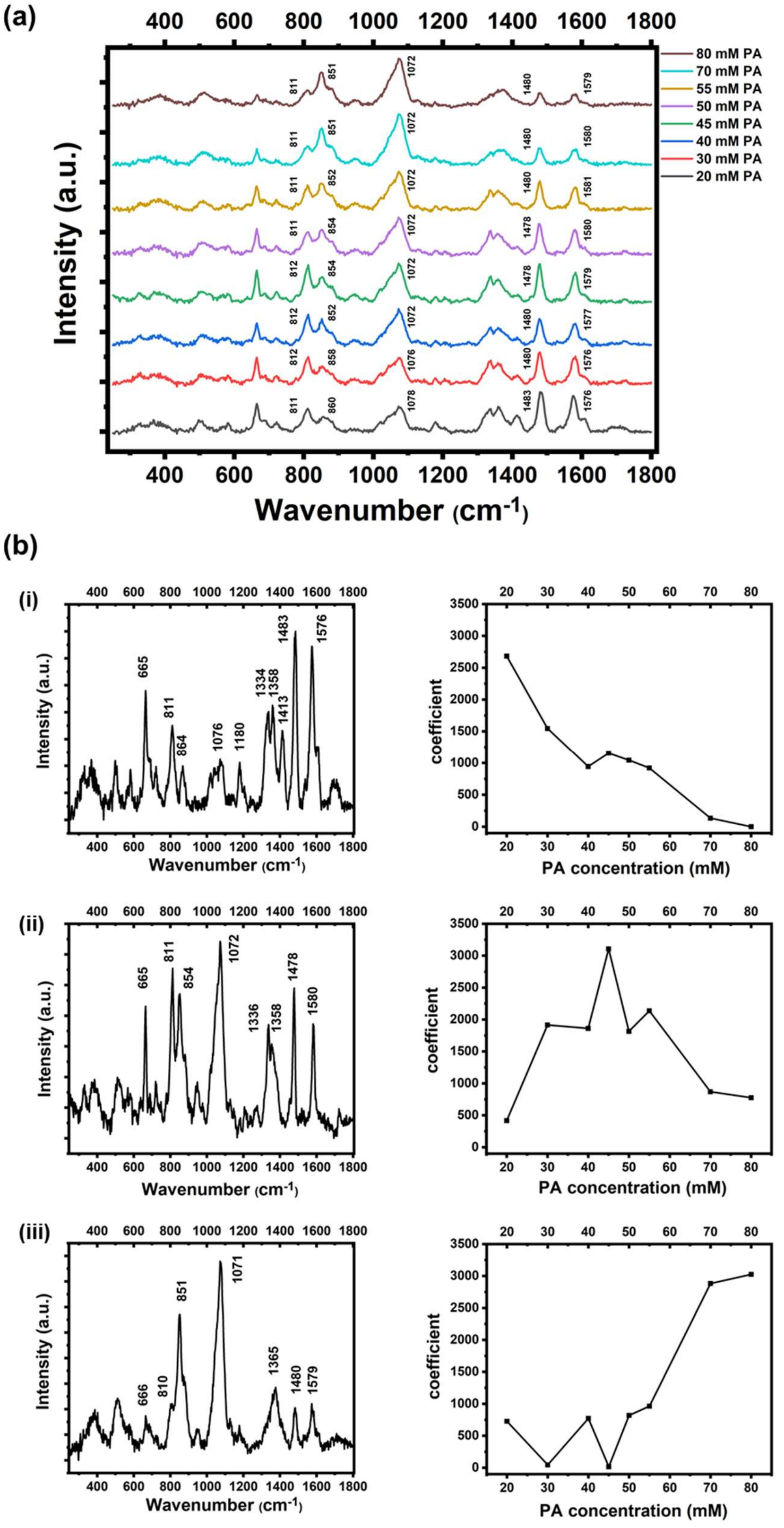


**Figure S16.** Singular value decomposition (SVD) was performed on (a) the spectral data set of varied PA concentration (20–80 mM). It indicated that three components

predominate the result. Multivariate curve resolution-alternative least squares (MCR-ALS) analysis was then applied using a non-negative matrix factorization algorithm, considering the three independent vectors in the spectrum and concentration dependence (Ando and Hamaguchi 2014). The MCR-ALS procedure was iterated 10000 times without setting penalty terms of the L1 and L2 norms.

(b) MCR-ALS analysis reveals the three concentration-dependent regimes corresponding to PA concentrations (i) <30 mM, (ii) 30-55 mM, and (iii) >70 mM. Below 30 mM, the Raman band at 1576 cm$^{-1}$ is stronger than that of the higher-concentration components (which correspond to 1585 cm$^{-1}$ in the main dataset), indicating that GMP predominantly exists in a monomeric state rather than forming G-tetrads. Additionally, the bands at 811 and 864 cm$^{-1}$ correspond to hydrogen-bonded and free PA molecules, respectively. Both peaks are detected within the 20–30 mM range, suggesting that hydrogen-bond formation begins in this region. The intensity of the 811 cm$^{-1}$ band increases further at 30–50 mM, indicating that hydrogen bonding becomes more pronounced within this range. Meanwhile, the 1483 cm$^{-1}$ band shifts to 1478 cm$^{-1}$ within the 30–55 mM range.

As the concentration enters the 30–55 mM range, the phosphate-related PA band at 1072 cm$^{-1}$ increases noticeably, reflecting the gradual rise in the molar fraction of PA. The 811/854 ratio also begins to decrease, indicating an increase in free PA molecules in the 30–55 mM range.

Above 70 mM, the PA phosphate band remains highly intense at 1071 cm$^{-1}$, and the 811/854 ratio decreases further, suggesting that hydrogen-bond formation does not increase at this stage, and that the additional PA exists primarily as free molecules. In contrast to the 30–55 mM range, the GMP-related peaks around 1480 and 1580 cm$^{-1}$ do not shift further, suggesting that the G-tetrad structure is fixed and does not change

with PA concentration beyond this range.

## Supplementary Materials and Methods

### Chemicals

Guanosine 5’monophosphate disodium salt hydrate (5’-GMP) was obtained from Thermo Fisher Scientific Inc. 45% (w/w) potassium hydroxide (KOH) solution was provided by Alfa Aesar. 50% (w/w) phytic acid (PA) solution was purchased from Sigma-Aldrich. Hydrochloric acid (HCl) solution (1 M) and 85% (w/w) ortho-phosphoric acid ($H_3PO_4$) solution were purchased from Honeywell. Potassium chloride (crystal; KCl) was supplied by J.T. Baker.

### Preparation of acid-mediated guanosine-5’-monophosphate (5’-GMP) hydrogels

The 5'-GMP and KCl stock solutions were prepared at 1 M and 1.459 M, respectively, in $ddH_2O$. The KOH solution (45% w/w) was diluted with $ddH_2O$ to become 1.4592 M. HCl, $H_3PO_4$, and PA aqueous solutions were prepared at 1 M by $ddH_2O$. For PA-mediated 5'-GMP self-assembly, potassium ion ($K^+$ sources either from $KCl_{(aq)}$ or $KOH_{(aq)}$), 5'-GMP, and PA were added sequentially, with concentrations adjusted to the desired concentrations (**Table S3**-**S5**) to investigate their respective concentration effects on gelation. Different acids (HCl, $H_3PO_4$, or PA)-mediated 5'-GMP assembly was also accordingly examined at pH3.

**Table S3.** Hydrogels prepared at different [PA]

| **[$K^+$] (KCl or KOH) (mM)** | **364.8** | | | | | | | |
|---|---|---|---|---|---|---|---|---|
| **[GMP] (mM)** | 100 in KCl system / 60 in KOH system | | | | | | | |
| **[phytic acid] (mM)** | 0 | 5 | 15 | 30 | 45 | 60 | 75 | 90 |

**Table S4.** Hydrogels prepared at different [GMP]

| | | | | | | | |
|---|---|---|---|---|---|---|---|
| **[K$^+$] (KCl or KOH) (mM)** | | | | **364.8** | | | |
| **[GMP] (mM)** | 0 | 20 | 40 | 60 | 80 | 100 | 120 |
| **[phytic acid] (mM)** | | | | 60 | | | |

**Table S5.** Hydrogels prepared at different [K$^+$]

| **[K$^+$] (KCl or KOH) (mM)** | **0** | **100** | **200** | **300** | **364.8** | **400** | **500** |
|---|---|---|---|---|---|---|---|
| **[GMP] (mM)** | 100 in KCl system / 60 in KOH system | | | | | | |
| **[phytic acid] (mM)** | | | | 60 | | | |

**Raman spectroscopy (532 nm confocal Raman microspectrometer setup)**

Details of the experimental setup has been described elsewhere (Chang, Okajima et al. 2014). A schematic illustration of the 532 nm confocal Raman setup is shown in **Figure S17**. The signal accumulation of each single Raman spectrum was 40 seconds, repeated 10 times at 532 nm laser excitation (average power = 40 mW). Regarding the data processing, the low wavenumber region (<250 cm$^{-1}$) and fingerprint region (250-2000 cm$^{-1}$) Raman spectra of all samples were firstly selected for intensity normalization at 1640 cm$^{-1}$ (HOH bending of solvent $H_2O$), and the solvent $H_2O$ was subtracted after normalization. Next, after masking the range of feature peaks by IGOR Pro (v7.08) software, the baseline was subtracted upon polynomial fitting. High wavenumber (>2000 cm$^{-1}$) Raman measurements were plotted as raw data without any normalization and baseline correction. As the CCD pixel index was used for the horizontal axis in raw spectral data, the atomic emission spectra (Ne lamps) were performed for Raman shift calibration.

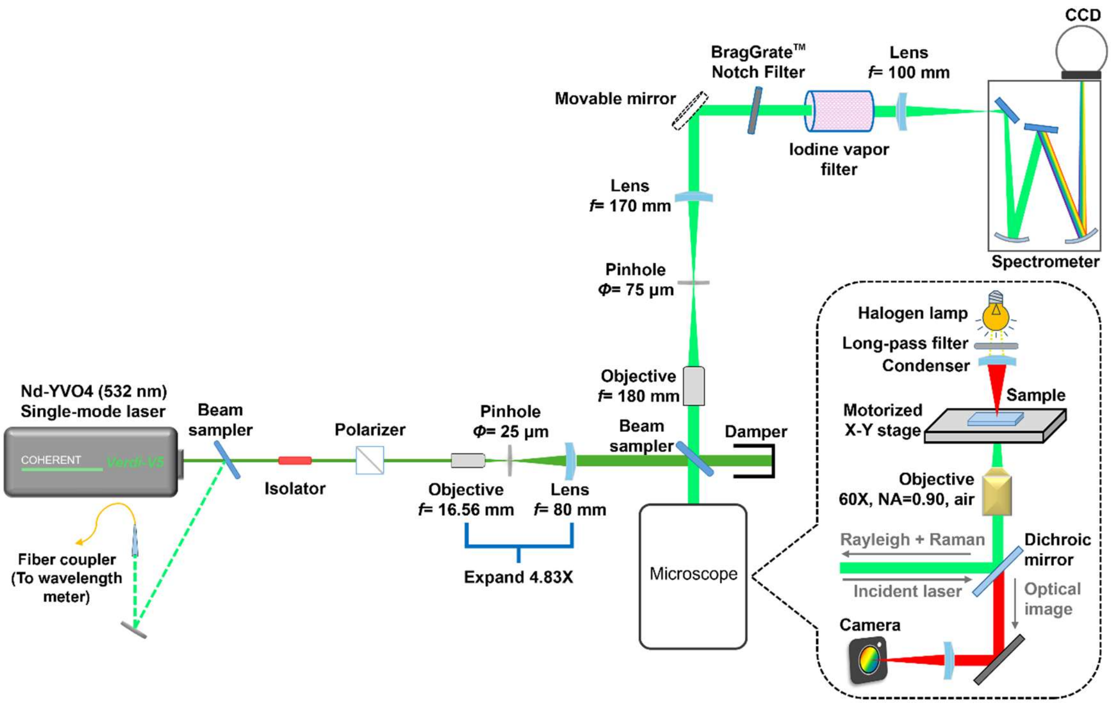


**Figure S17.** Schematic diagram of the confocal Raman spectrometer (adapted from <u>*Chang, Okajima et al. 2014*</u> with permission from the Royal Society of Chemistry).

Raman difference spectrum was applied by subtracting the spectrum of the reference product from the spectrum of the sample to detect and highlight the changes in the sample. The general formula:

$$\text{Difference} = S - c \times R \qquad \textbf{eq. S1}$$

*S* represents the Raman spectrum of the sample, c denotes a specific coefficient, and *R* is the Raman spectrum of the reference. The designation of coefficient c depends on whether the purpose is to explore any differences except for or including the intensity. To identify newly appeared or disappeared peaks, the coefficient should be chosen to minimize the difference spectrum. If it is intended to detect any difference, the coefficient should be set as 1.

The Raman band shift is due to the vibrational energy changes of the molecule,

and the energy is directly related to the vibrational frequency $v$. The atomic vibrations frequency $v$ can be explained with the Schrödinger equation for harmonic oscillator, and for a di-atomic molecule the following equation is applied:

$$v = \frac{1}{2\pi}\sqrt{\frac{k}{\mu}} \qquad \textbf{eq. S2}$$

$k$ denotes the force constant, while $\mu$ is the reduced mass of the molecule.

The force constant $k$ of a molecular bond is a measure of the bond strength and is related to both bond order and bond length. Bond order is the number of chemical bonds between paired atoms. Higher bond order (e.g., double or triple bonds) indicates stronger bonds because multiple bonding interactions between atoms result in stiffer bonds supporting higher force constants. The relationship between force constant $k$ and bond order can be expressed as:

$$k \propto \text{bond order} \qquad \textbf{eq. S3}$$

Alternatively, shorter bond lengths indicate stronger bonds because atoms with shorter bonds are more tightly bonded and require more energy to move them from the equilibrium position, corresponding to a higher force constant. Empirically, the force constant $k$ tends to be inversely dependent on the bond length $r$:

$$k \propto \frac{1}{r^3} \qquad \textbf{eq. S4}$$

Generally, higher-order and shorter bonds tend to have higher force constants, indicating stronger bonds, which refer to the higher frequency in Raman spectra.

**Rheological fitting**

A springpot was used in modelling and to capture the rheological response of the system (Bonfanti, Kaplan et al. 2020). The fitting results were summarized in **Table S1-S2**. Overall, good to acceptable fitting quality was achieved in most of our experimental conditions, with $R^2$ ranging from 0.85 to 0.99. Among the fitted parameters, the

fractional exponent β is the most informative descriptor. A value of β approaching 0 reflects elastic-dominated behavior, whereas a value of β approaching 1 indicates viscous dominance. As shown in fitting results, β remained within the range of 0.02–0.33 across samples, indicating solid-like, weak-power-law viscoelasticity. Alternatively, the prefactor C scales primarily with modulus magnitude and is more sensitive to structural stiffness than to dynamic response. C is thus interpreted as an amplitude factor rather than a kinetic indicator.

## Reference

Ando, M. and H. O. Hamaguchi (2014). "Molecular component distribution imaging of living cells by multivariate curve resolution analysis of space-resolved Raman spectra." J. Biomed. Opt. **19**(1): 011016.

Bonfanti, A., J. L. Kaplan, G. Charras and A. Kabla (2020). "Fractional viscoelastic models for power-law materials." Soft Matter **16**(26): 6002-6020.

Chang, C.-F., H. Okajima, H.-o. Hamaguchi and S. Shigeto (2014). "Imaging molecular crystal polymorphs and their polycrystalline microstructures in situ by ultralow-frequency Raman spectroscopy." Chem. Commun. **50**(85): 12973-12976.
